# Supplementary material for: Janus electronic state of supported iridium nanoclusters for sustainable alkaline water electrolysis
Source: Nat Commun. 2024 Apr 2;15:2851. doi: 10.1038/s41467-024-47045-6 (PMC10987502; doi:10.1038/s41467-024-47045-6)
Supplement: Supplementary file 1 — Supplementary Information [file 41467_2024_47045_MOESM1_ESM.pdf]

Supplementary Information

Janus electronic state of supported iridium nanoclusters for sustainable alkaline water electrolysis

Yaoda Liu[1], Lei Li[1], Li Wang[2], Na Li[2], Xiaoxu Zhao[3], Ya Chen[1], Thangavel Sakthivel[4], & Zhengfei Dai[1]*

[1] State Key Laboratory for Mechanical Behavior of Materials, Xi'an Jiaotong University, Xi'an 710049, P. R. China

[2] State Key Laboratory for Powder Metallurgy, Central South University, Changsha 410083, P. R. China

[3] School of Materials Science and Engineering, Peking University, Beijing100871, P. R. China

[4] Department of Chemical Engineering, Kumoh National Institute of Technology, Gyeongbuk 39177, South Korea

*email: sensdai@mail.xjtu.edu.cn

This file includes:

Supplementary Notes 1 to 2

Supplementary Figures 1 to 65

Supplementary Tables 1 to 11

Supplementary References 1 to 74

Table of Contents

1. Supplementary Notes S3

2. Supplementary Figures.. S6

3. Supplementary Tables S71

4. Supplementary References S82

Supplementary Notes

Supplementary Note 1. DFT Calculations of OER Performance

Under alkaline conditions, the process of OER reaction can be described by the following four steps,

$$OH^{-}+*\longrightarrow *OH+e^{-} \quad (1)$$

$$*OH+OH^{-}\longrightarrow *O+H_2O+e^{-} \quad (2)$$

$$*O+OH^{-}\longrightarrow *OOH+e^{-} \quad (3)$$

$$*OOH+OH^{-}\longrightarrow O_2+*+H_2O+e^{-} \quad (4)$$

in which * denotes the pure surface without adsorbed adsorbate, *O, *OH, *OOH denote the surface system with adsorbed intermediate O, OH, and OOH, respectively. The Gibbs free energy variations of the above four reaction steps are represented as ΔG_1, ΔG_2, ΔG_3, and ΔG_4, respectively.

$$\Delta G_1=G_{*OH}+(\frac{G_{H_2}}{2}-0.0592pH)+eU-G_{*}-G_{H_2O} \quad (5)$$

$$\Delta G_2=G_{*O}+(\frac{G_{H_2}}{2}-0.0592pH)+eU-G_{*OH} \quad (6)$$

$$\Delta G_3=G_{*OOH}+(\frac{G_{H_2}}{2}-0.0592pH)+\mathrm{eU}-G_{*O}-G_{H_2O} \quad (7)$$

$$\Delta G_4=G_{O_2}+G_{*}+\left(\frac{G_{H_2}}{2}-0.0592pH\right)+\mathrm{eU}-G_{*OOH} \quad (8)$$

U is the applied electrode potential. The standard electrode potential is 0.401 eV at pH = 14 under alkaline conditions, and the Gibbs free energy change of the determination step is $G^{OER}=\max(\Delta G_1, \Delta G_2, \Delta G_3, \Delta G_4)$, and the theoretical calculated overpotential is $\eta=G^{OER}-0.401$. The adsorption sites are shown in Supplementary Fig. 47-52 and the calculation results are shown in Supplementary Table 6.

Supplementary Note 2. DFT Calculations of HER Performance

The adsorption behavior of H_2O on the electrocatalyst surface is a crucial factor for water splitting. In our calculations, the adsorption strength of H_2O on the surface of Ir (111), $NiPS_3$, and Ir/$NiPS_3$ was considered.

The adsorption energy ΔE_{H_2O} can be calculated by Equation 9. The adsorption sites are shown in Supplementary Fig. 53, and the calculation results are shown in Supplementary Table 7.

$$\Delta E_{H_2O}=E_{DFT}^{H_2O^*}-E_{DFT}^{*}-E_{DFT}^{H_2O} \quad (9)$$

Where $E_{DFT}^{H_2O^*}$, E_{DFT}^{*} and $E_{DFT}^{H_2O}$ represent total energies of the surface with an adsorbed H_2O molecule, the surface, and a H_2O molecule, respectively.

The key reaction steps of HER under alkaline conditions can be described as:

Step1: H_2O + e^- + cat. → H*-cat. + OH^- (Volmer step)

Step2: 2H*-cat. → H_2↑ (Tafel step)

Step3: H*-cat. + H_2O + e^- → cat. + OH^- + H_2↑ (Heyrovsky step)

At the equilibrium potential of HER, the free energies of Step 1 and 3 should be the same. Under this assumption, calculating the exact free energy of OH^- in solution is avoided. In our calculations, four main stages are considered: initial state, activated water (OH-H) adsorption, H* intermediate adsorption, H_2 formation and desorption. Given this, the energies for different stages at the reduction potential U=0 V vs. RHE is calculated as follows:

$$E_0=E_{cat.}+E_{H_2O} \quad (10)$$

$$E_1=E_{cat.\text{-}(H\text{-}OH)} \quad (11)$$

$$E_2=E_{cat.\text{-}H*}+E_{OH^-} \quad (12)$$

$$E_3=E_{cat.}+E_{OH^-}+\frac{1}{2}E_{H_2} \quad (13)$$

Herein, based on many previous studies, the $\Delta E_{H\text{-}OH}$ value is used as the activity descriptor for the Volmer step. The species H-OH is the ground state for activated water adsorption. ΔE_{H^*} values are used as activity descriptors for Tafel steps or Heyrovsky steps:

$$\Delta E_{H\text{-}OH}=E_1-E_0=E_{cat.\text{-}(H\text{-}OH)}-E_{cat.}-E_{H_2O} \quad (14)$$

$$\Delta E_{H^*}=E_2-E_3=E_{cat.\text{-}H*}-E_{cat.}-\frac{1}{2}E_{H_2} \quad (15)$$

$$E_0=E_3 \quad (16)$$

Furthermore, the Gibbs free energies for hydrogen adsorption in the HER process were corrected at 298.15 K, ΔG_{H^*} can be evaluated by Equation 17:

$$\Delta G_{H^*}=\Delta E_{H^*}+\Delta E_{ZPE}-T\Delta S_H \quad (17)$$

where ΔE_{H^*}, ΔE_{ZPE} and $T\Delta S_H$ represent the differences of hydrogen adsorption energy, zero-point energy, and the entropy between adsorbed hydrogen and hydrogen in the gas phase, respectively. The calculation results are shown in Supplementary Table 8,9, and the adsorption sites are shown in Fig. 5l and Supplementary Fig. 54-57.

Supplementary Figures and Tables

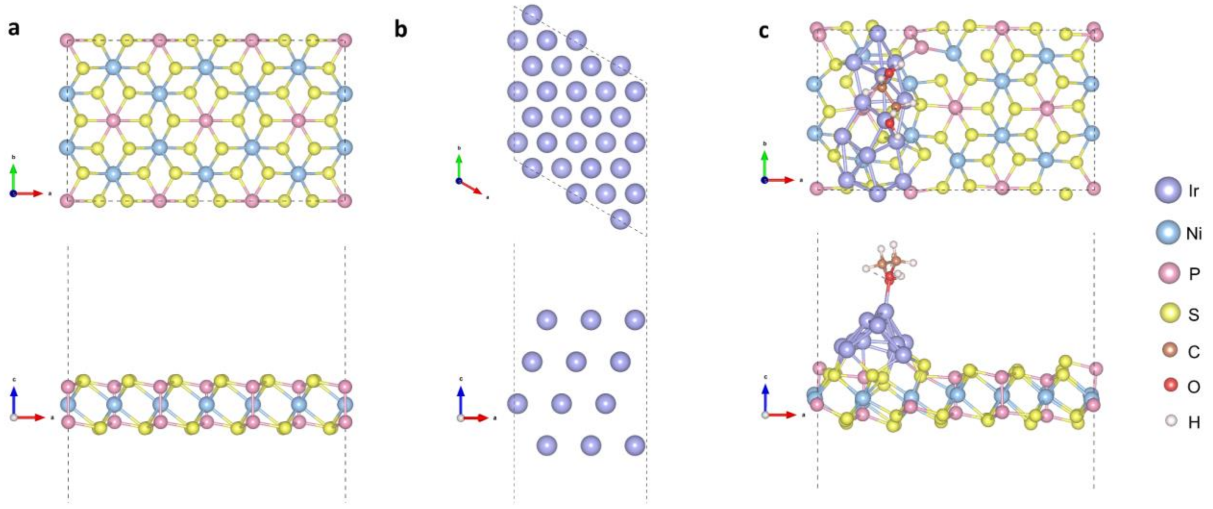

Supplementary Fig. 1. The atomic models of (a) $NiPS_3$, (b) Ir surface, (c) Ir/$NiPS_3$. The ligand effect was fully evaluated, and models with and without ethylene glycol ligands were constructed, respectively.

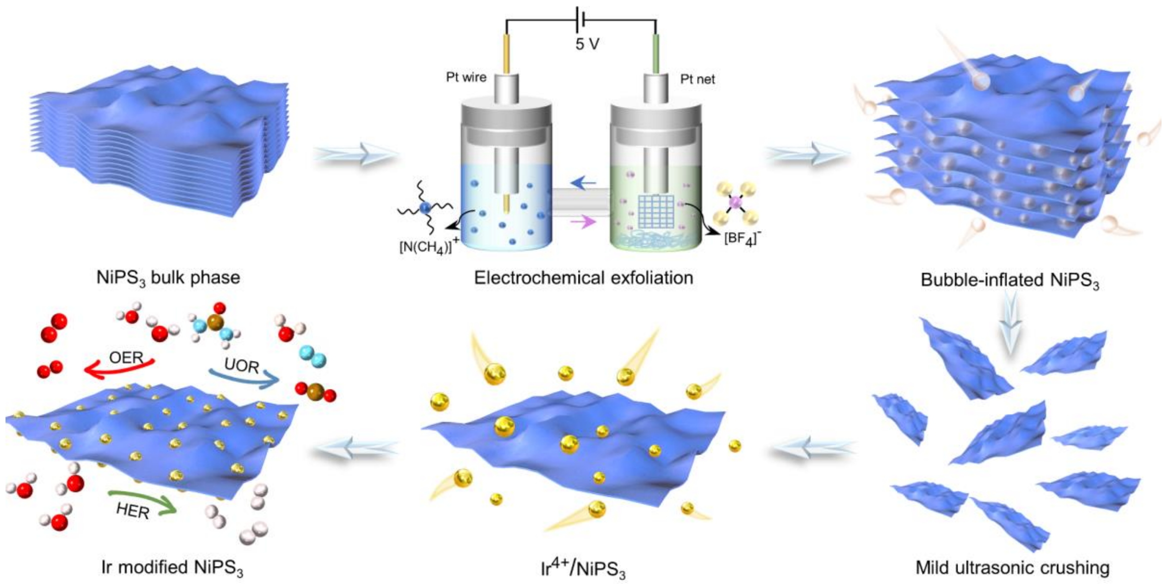

Supplementary Fig. 2. The synthetic scheme of Ir/$NiPS_3$.

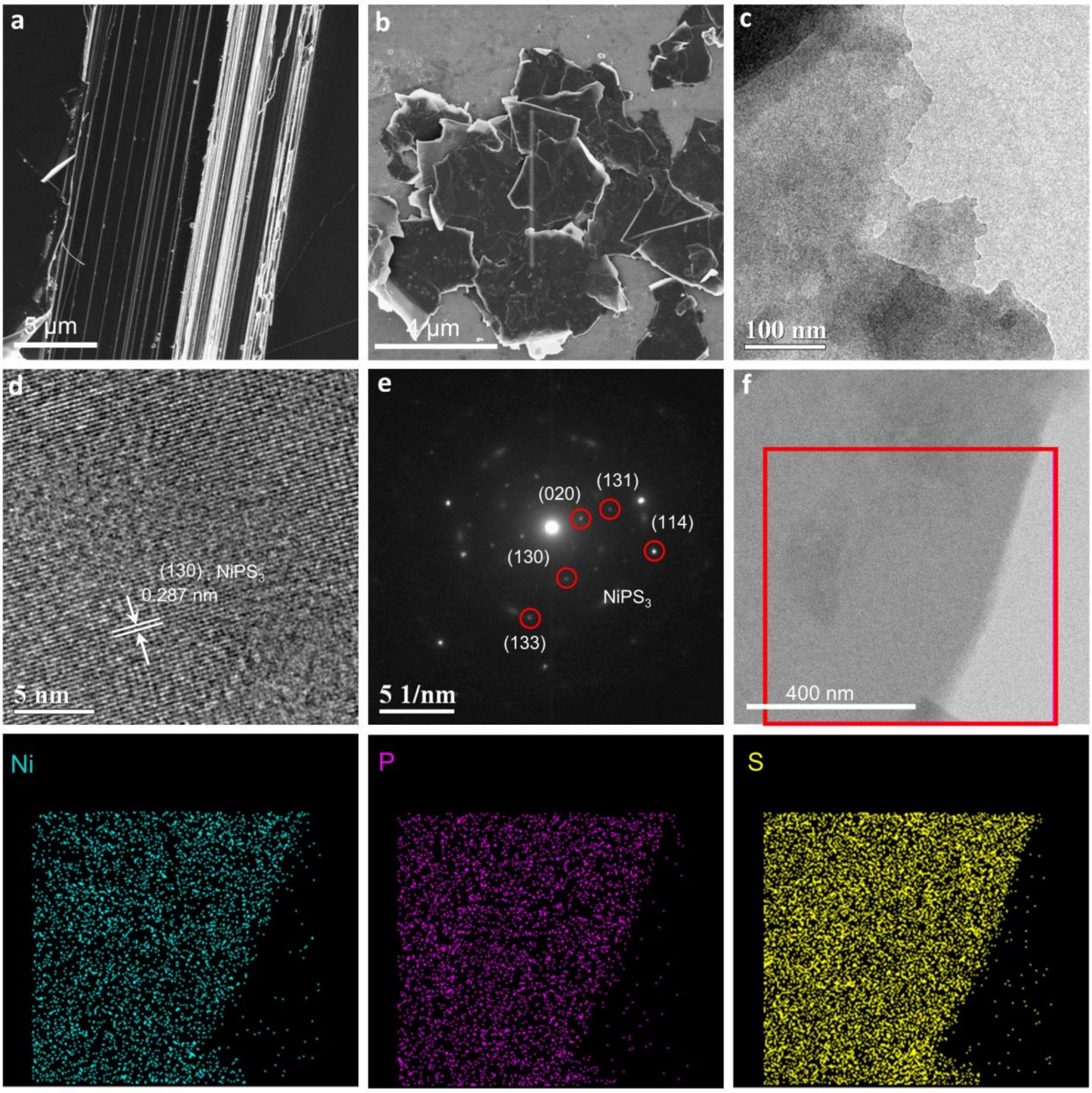

Supplementary Fig. 3. SEM images of (a) bulk $NiPS_3$ and (b) EE-$NiPS_3$. (c) TEM image, (d) HR-TEM image, (e) SAED pattern, and (f) EDS mapping of EE-$NiPS_3$.

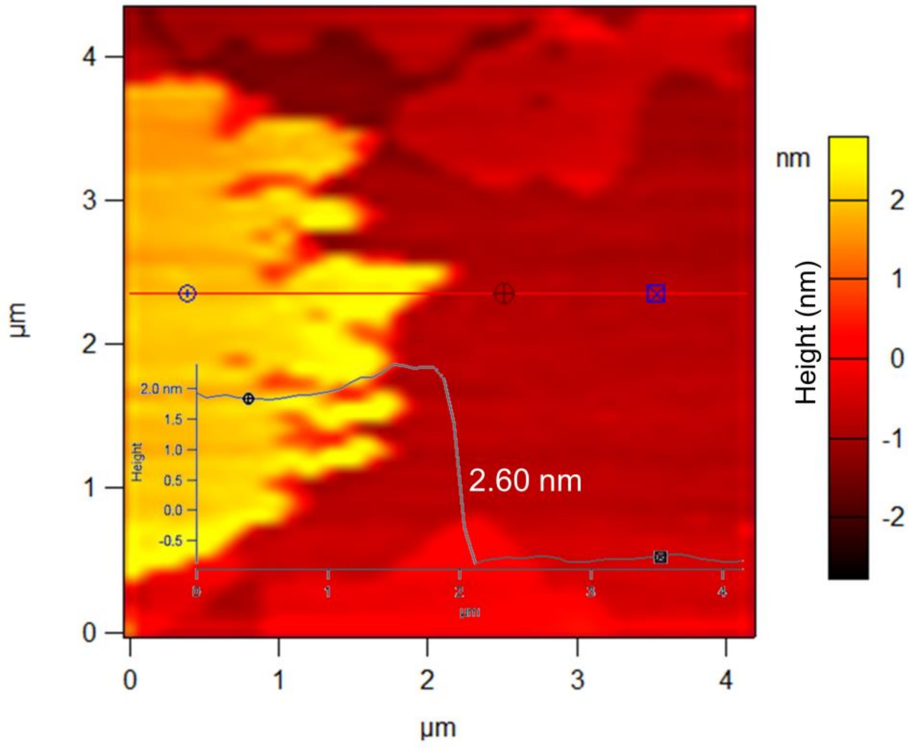

Supplementary Fig. 4. The AFM photograph of EE-$NiPS_3$ and corresponding thickness measurement results (inset).

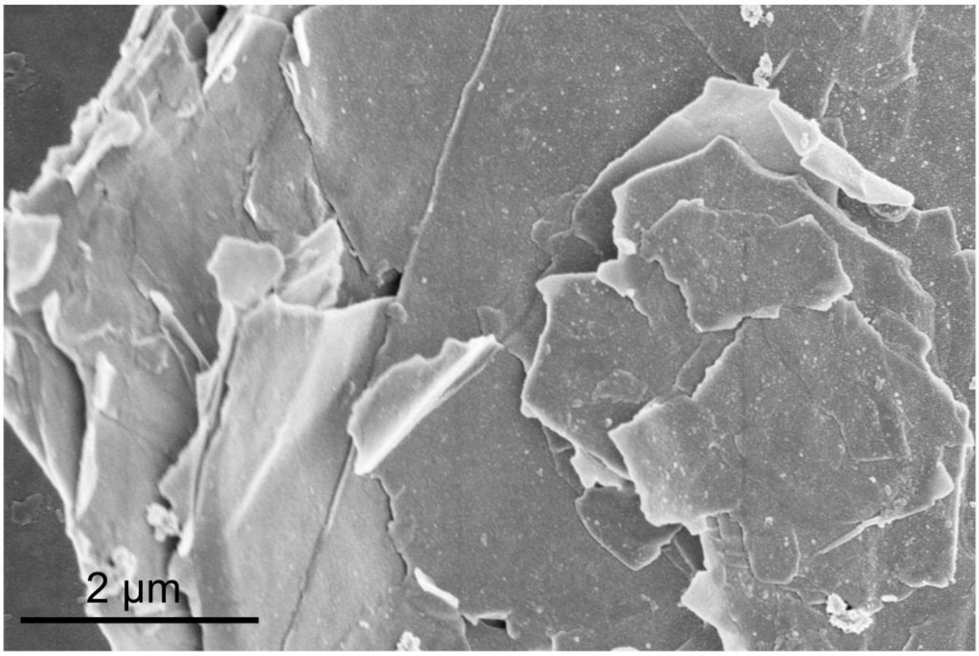

Supplementary Fig. 5. The SEM image of Ir/$NiPS_3$.

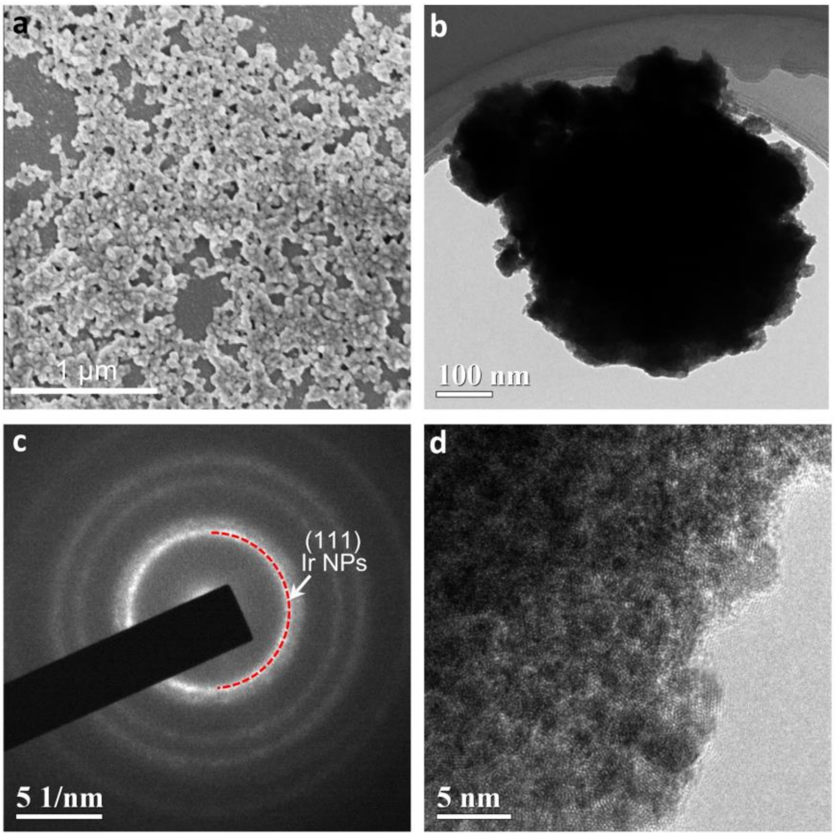

Supplementary Fig. 6. (a) SEM image, (b) TEM image, (c) SAED pattern, and (d) HR-TEM image of Ir NPs.

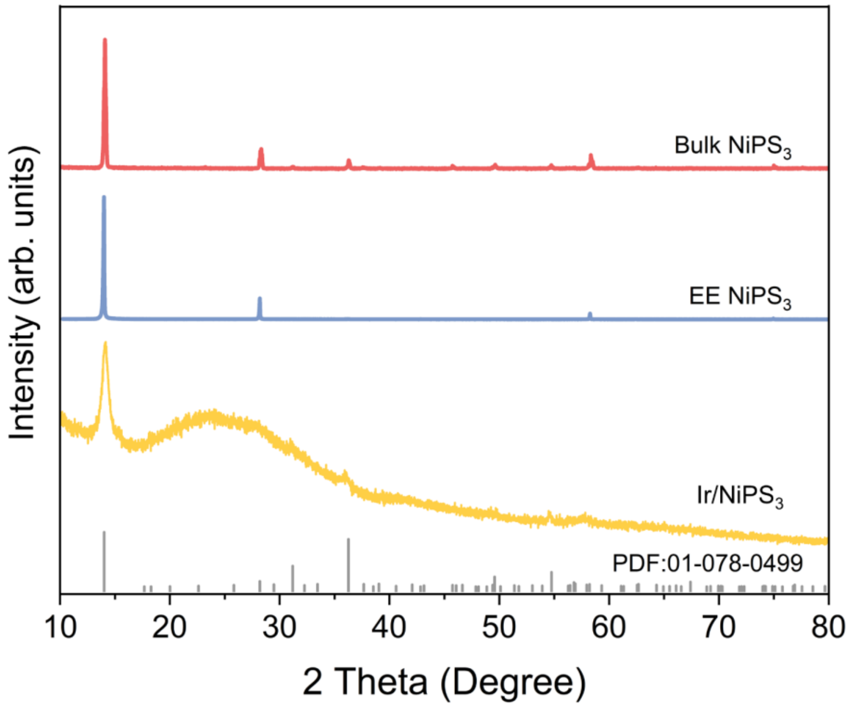

Supplementary Fig. 7. XRD patterns of bulk $NiPS_3$, EE-$NiPS_3$, and Ir/$NiPS_3$.

The series of peaks of bulk $NiPS_3$ matches well with the standard PDF card of $NiPS_3$ (JCPDS no. 01-078-0499). After electrochemical exfoliation to get EE-$NiPS_3$, only the (001), (002), and (004) crystal planes corresponding to the peaks at around 14.0°, 28.2°, 58.2° can be observed in the XRD pattern, indicating the successful exfoliation of bulk into few layers.

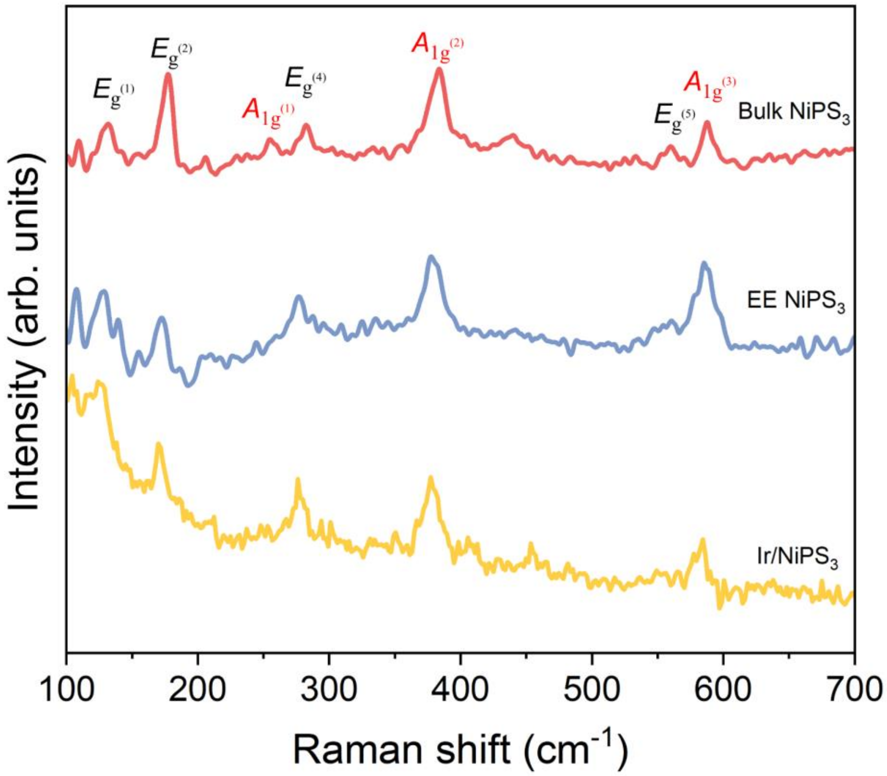

Supplementary Fig. 8. Raman spectra of bulk $NiPS_3$, EE-$NiPS_3$, and Ir/$NiPS_3$.

It shows the typical in-plane E_g (~130.8, 177.1, 283.2, and 560.9 cm^{-1}) and out-of-plane A_{1g} (~256.2, 383.4, and 588.5 cm^{-1}) phonon modes of $NiPS_3$[3, 4]. It is observed that the A_{1g} peaks of EE-$NiPS_3$ show red shifts after exfoliation from bulk phase, which is due to the weakening of interlaminar coupling as the thickness of $NiPS_3$ became thinner.

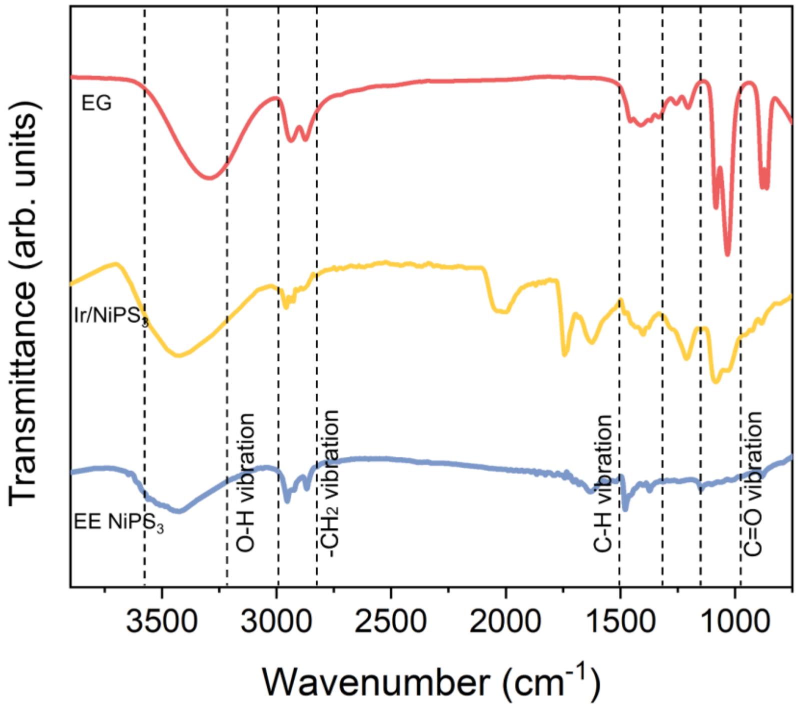

Supplementary Fig. 9. FT-IR spectra of EG, EE-$NiPS_3$, and Ir/$NiPS_3$.

The distinct peaks in several regions with wavenumbers ranging from large to small can be ascribed to the water O-H bending vibration, -CH_2 vibration, C-H vibration, and C-O vibration, respectively [5, 6, 7].

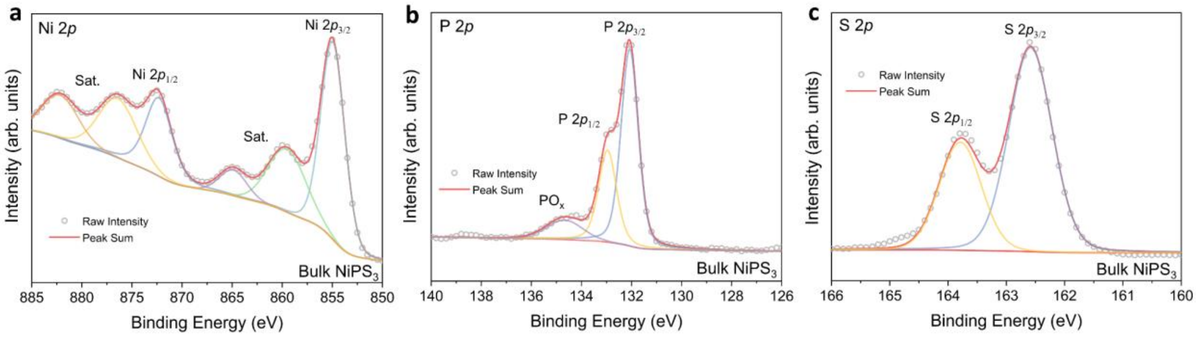

Supplementary Fig. 10. (a) Ni 2*p*, (b) P 2*p*, and (c) S 2*p* XPS spectra of bulk $NiPS_3$.

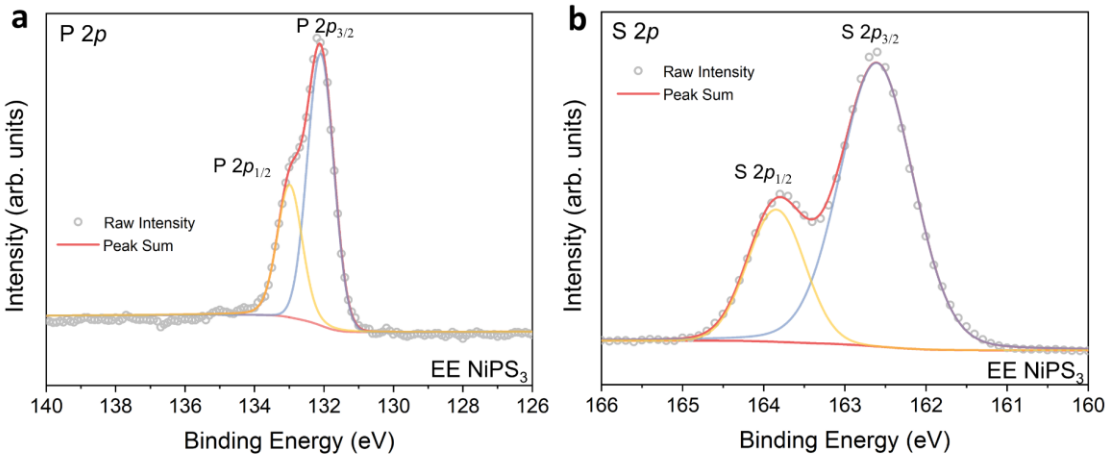

Supplementary Fig. 11. (a) P 2*p* and (b) S 2*p* XPS spectra of EE-$NiPS_3$.

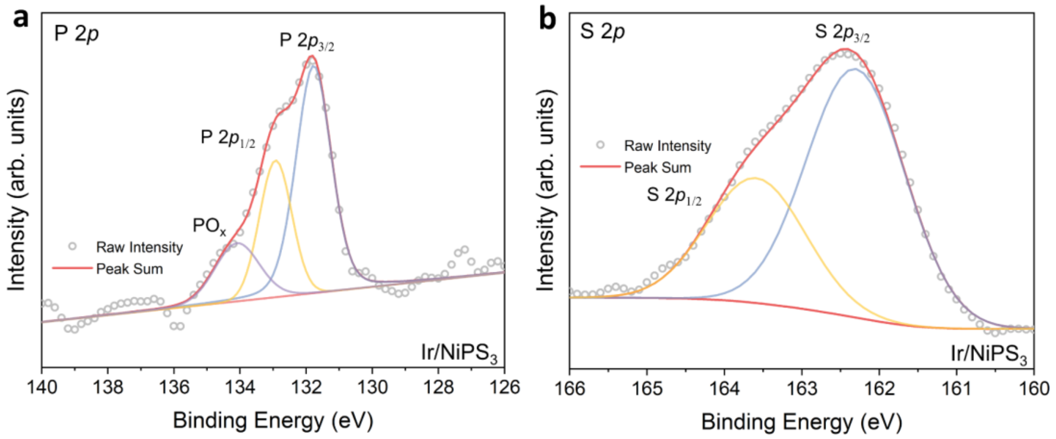

Supplementary Fig. 12. (a) P 2*p* and (b) S 2*p* spectra of Ir/$NiPS_3$.

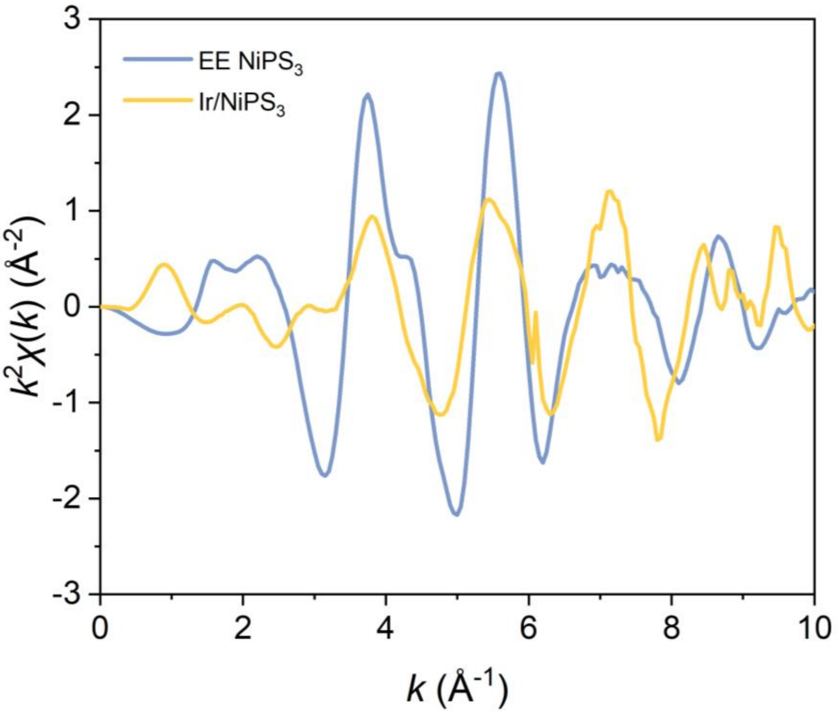

Supplementary Fig. 13. The *k*-space spectra of EE-$NiPS_3$ and Ir/$NiPS_3$ for Ni K-edge.

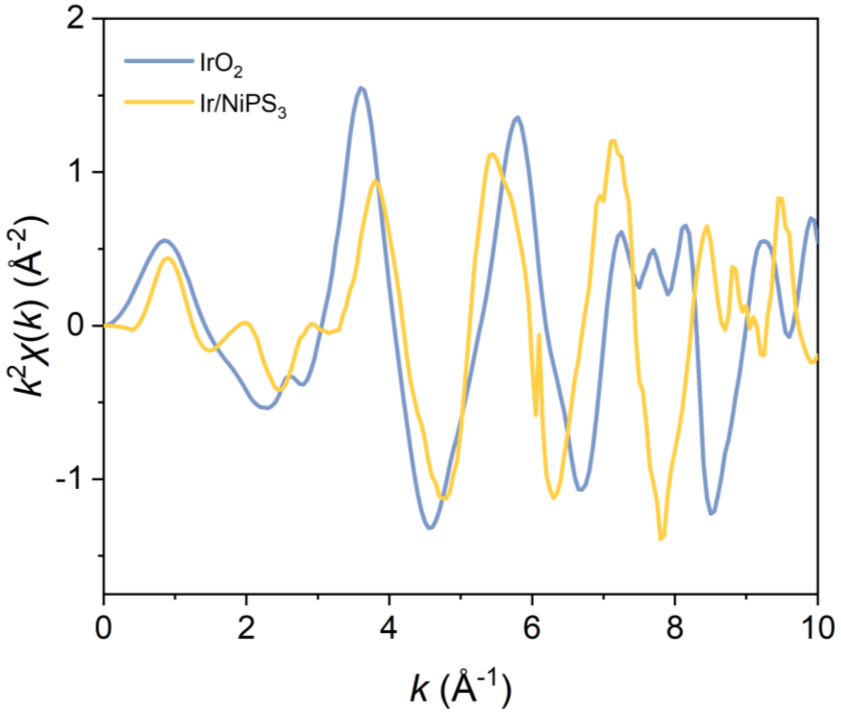

Supplementary Fig. 14. The *k*-space spectra of IrO_2 and Ir/$NiPS_3$ for Ir L_3-edge.

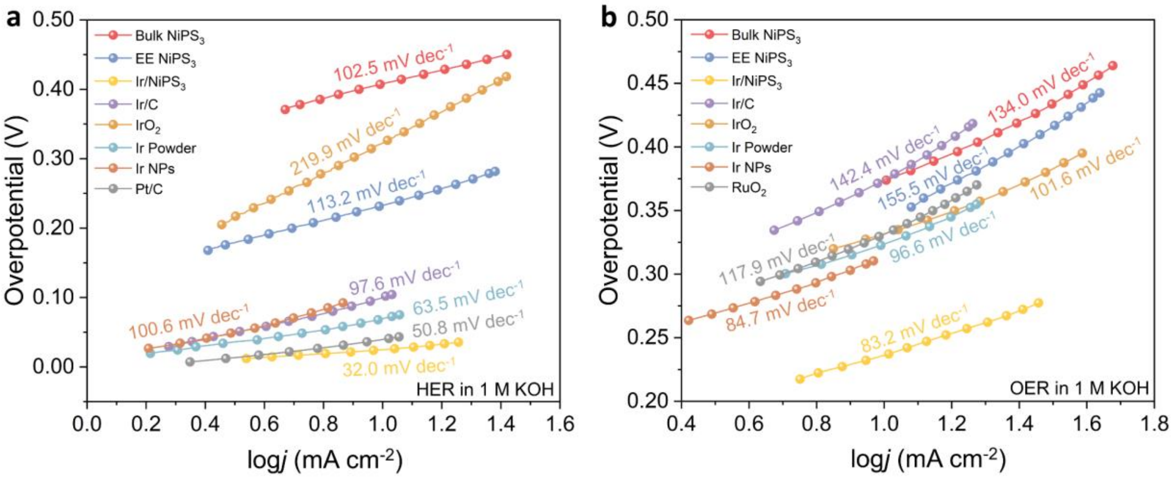

Supplementary Fig. 15. The Tafel plots for (a) HER and (b) OER in 1 M KOH.

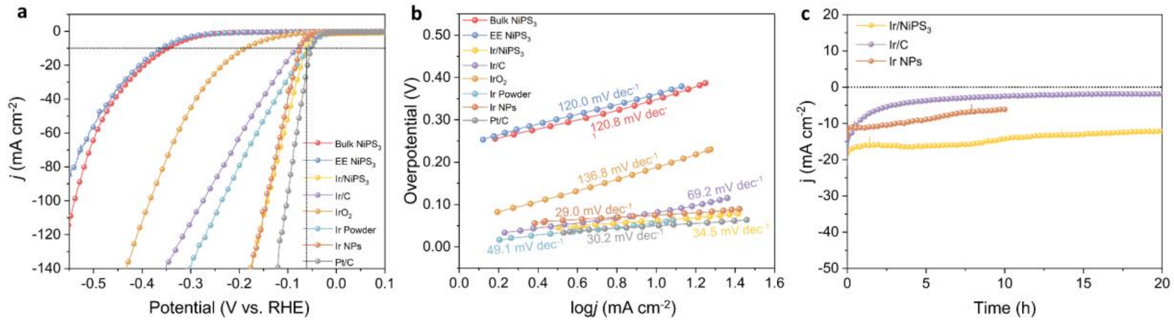

Supplementary Fig. 16. Electrocatalytic performance of samples in acid (0.5 M H_2SO_4) electrolyte for HER: (a) LSV curves, (b) Tafel plots, (c) Chronopotentiometry curves.

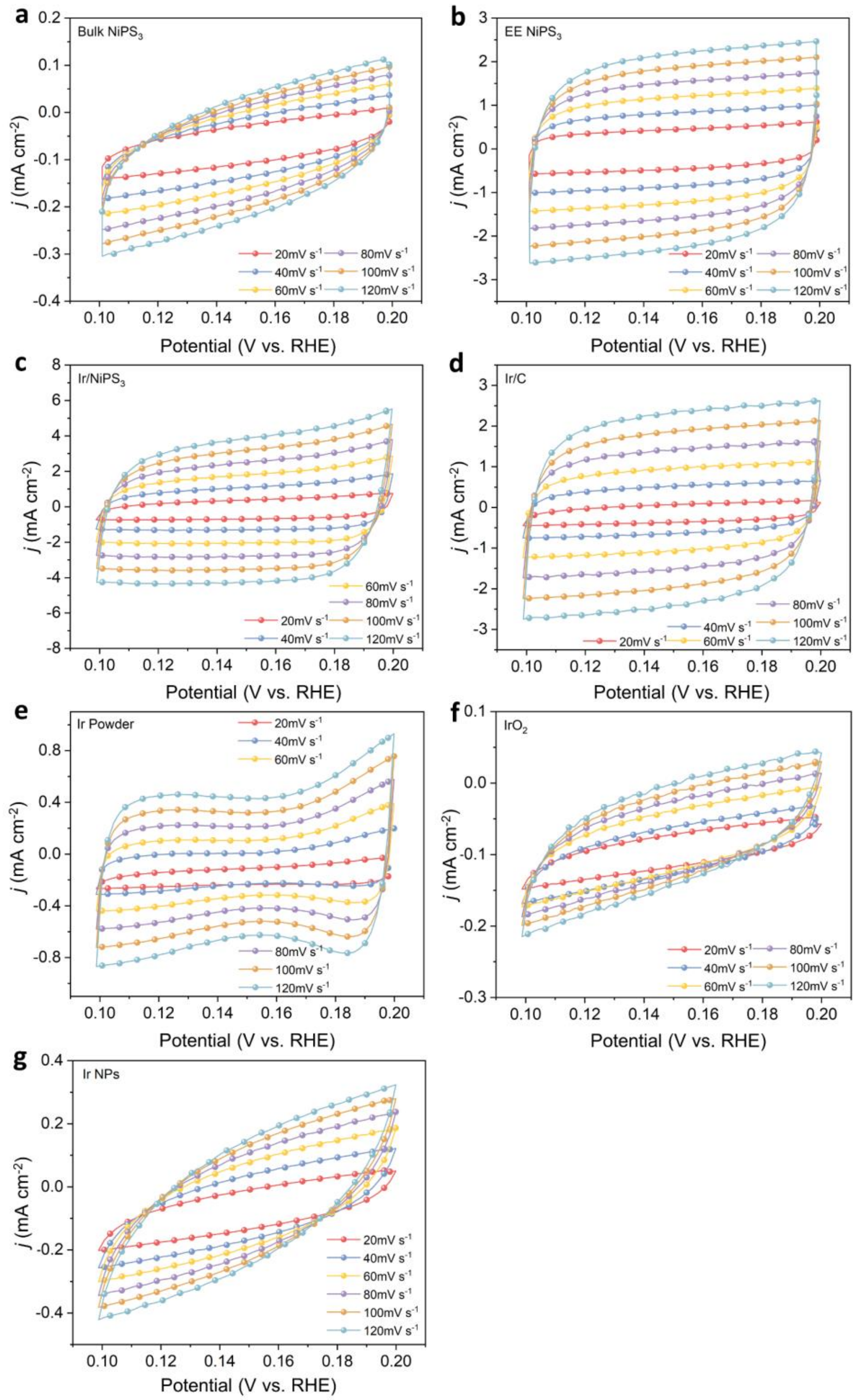

Supplementary Fig. 17. The current-potential curves of samples with different scan rates for HER process in 1 M KOH: (a) Bulk $NiPS_3$, (b) EE-$NiPS_3$, (c) Ir/$NiPS_3$, (d) Ir/C, (e) Ir powder, (f) IrO_2, and (g) Ir NPs.

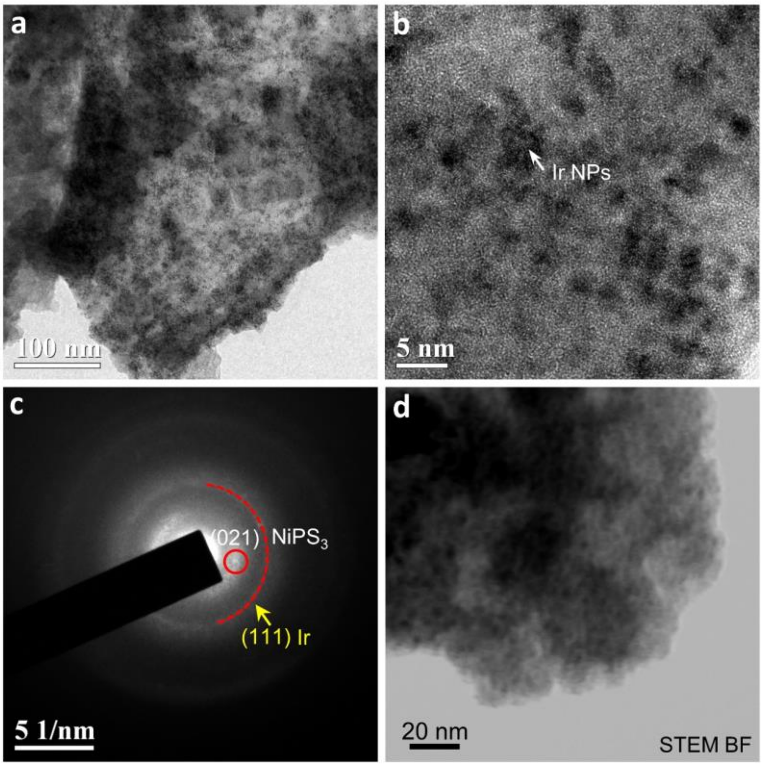

Supplementary Fig. 18. (a) TEM image, (b) HR-TEM image, (c) SAED pattern, and (d) STEM-BF image of Ir/$NiPS_3$ after HER cycling test in 1 M KOH.

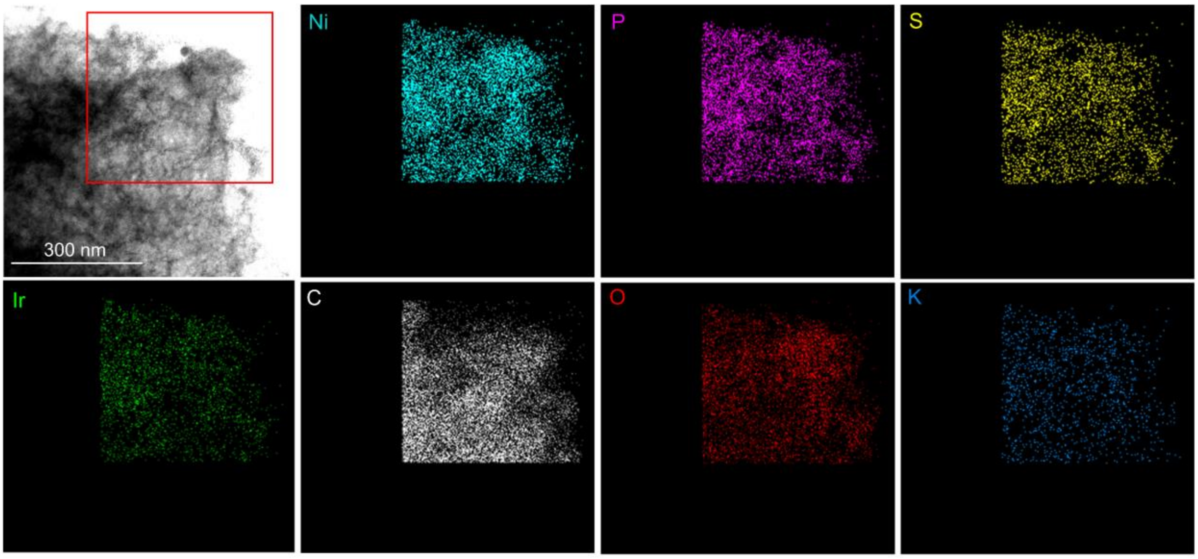

Supplementary Fig. 19. EDS mapping images of Ir/$NiPS_3$ after HER cycling test in 1 M KOH.

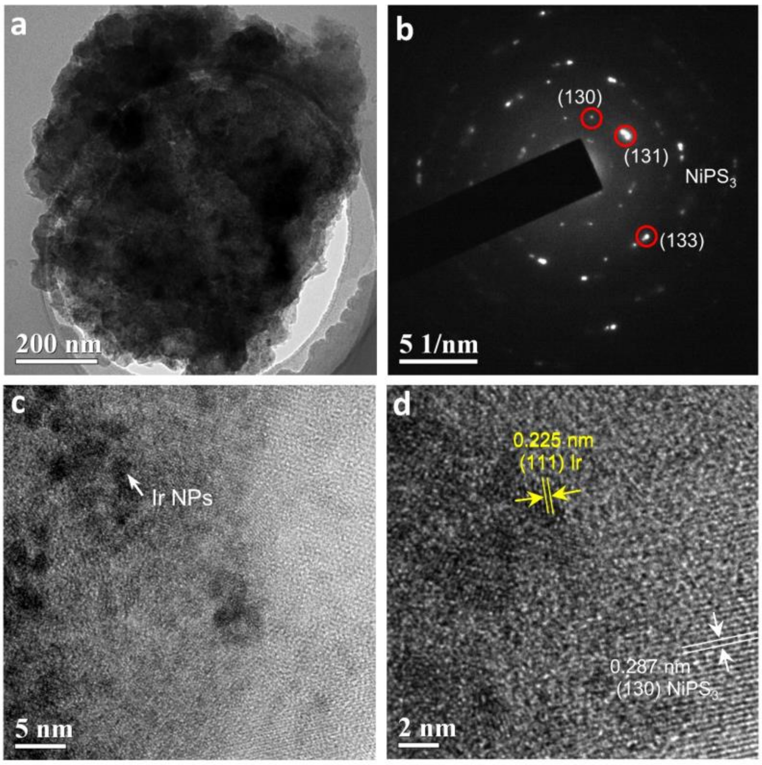

Supplementary Fig. 20. (a) TEM image, (b) SAED pattern, (c,d) HR-TEM images of Ir/$NiPS_3$ after HER cycling test in 0.5 M H_2SO_4.

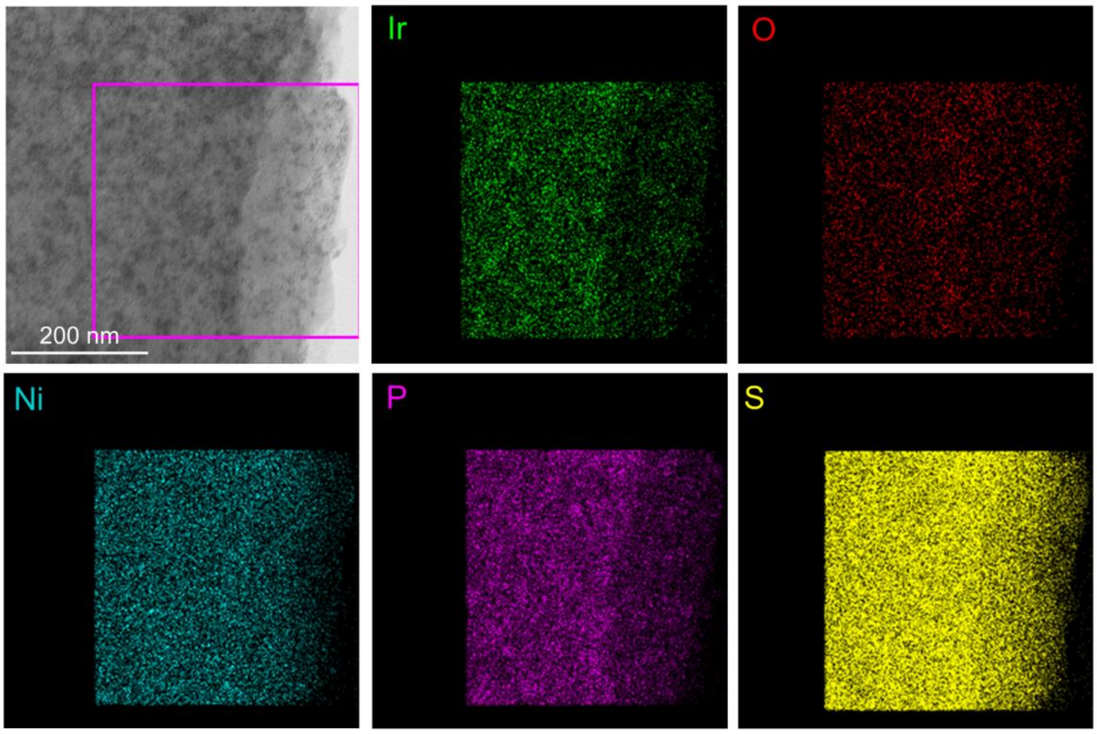

Supplementary Fig. 21. EDS mapping images of Ir/$NiPS_3$ after HER cycling test in 0.5 M H_2SO_4.

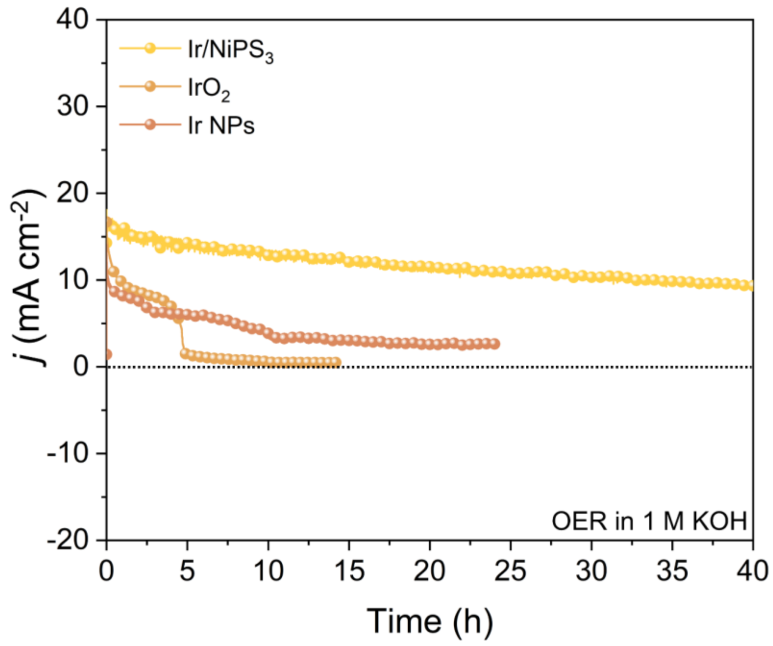

Supplementary Fig. 22. Chronopotentiometry curve for OER in 1 M KOH.

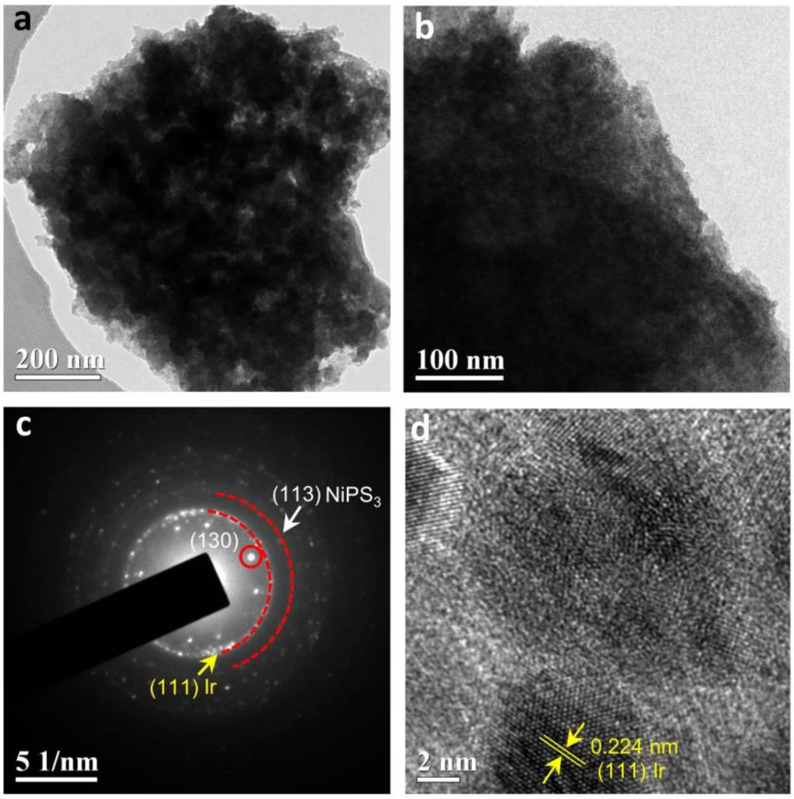

Supplementary Fig. 23. (a,b) TEM images, (c) SAED pattern, (d) HR-TEM image of $Ir/NiPS_3$ after OER cycling test in 1 M KOH.

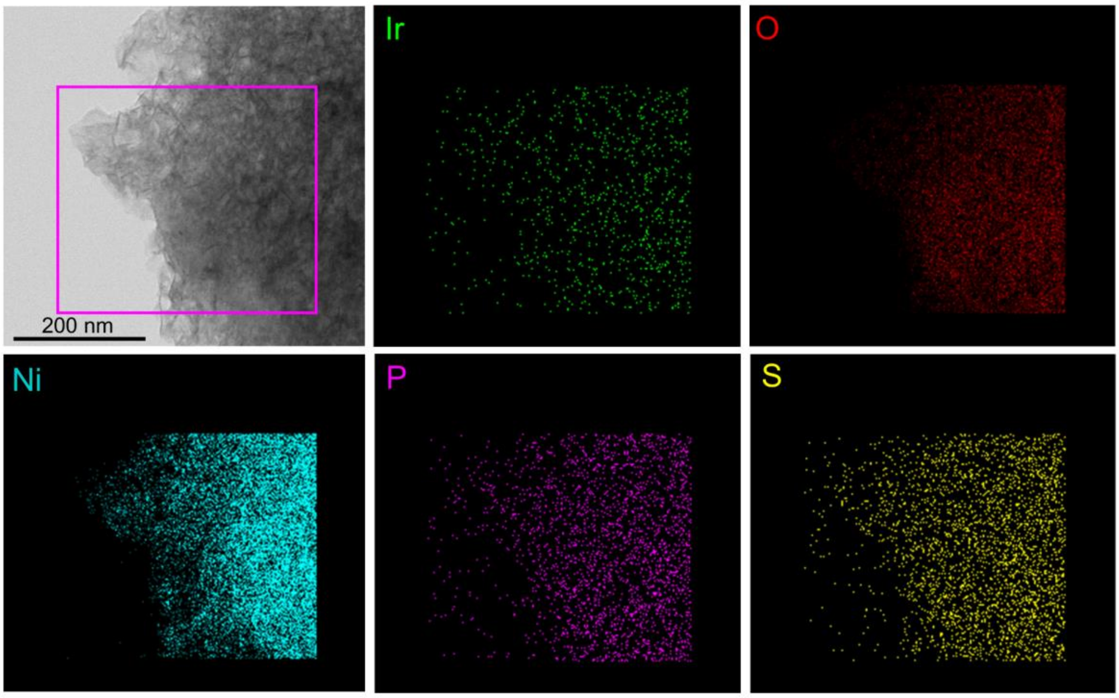

Supplementary Fig. 24. EDS mapping images of Ir/$NiPS_3$ after OER cycling test in 1 M KOH.

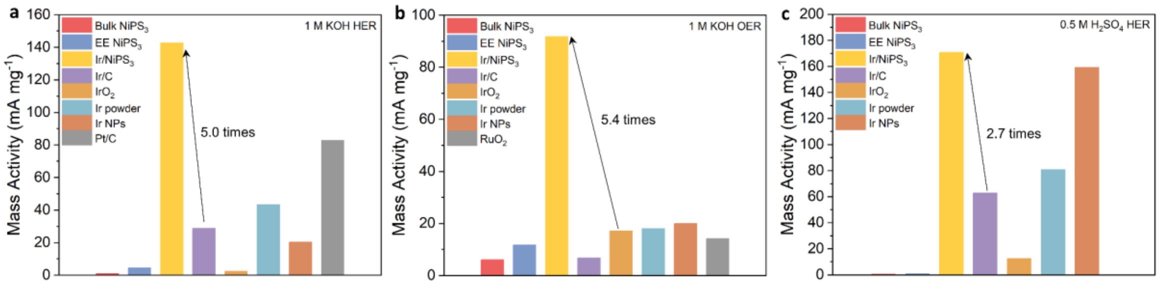

Supplementary Fig. 25. Mass activities of samples (a) for HER in 1 M KOH, (b) for OER in 1 M KOH, and (c) for HER in 0.5 M H_2SO_4, respectively.

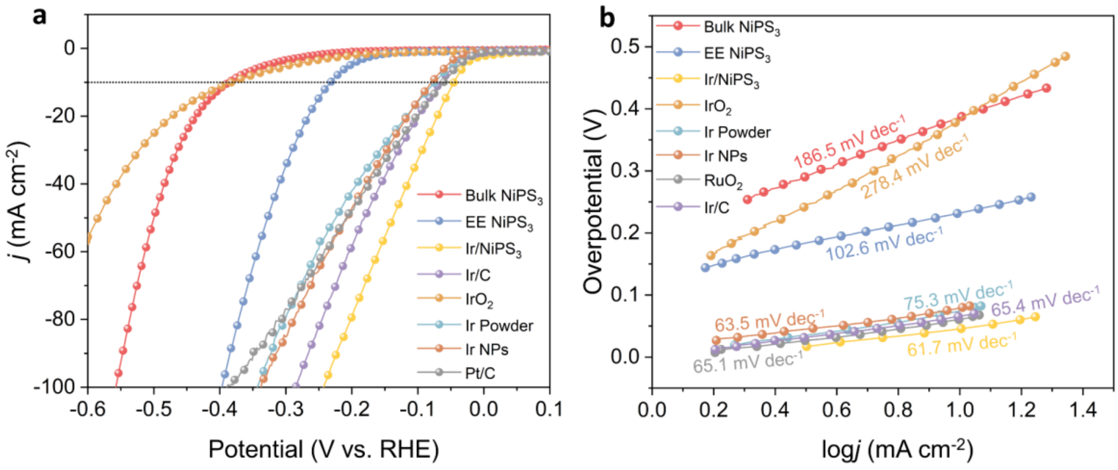

Supplementary Fig. 26. Electrocatalytic performance of samples in 1 M KOH + 0.5 M urea electrolyte for HER: (a) LSV curves, (b) Tafel plots.

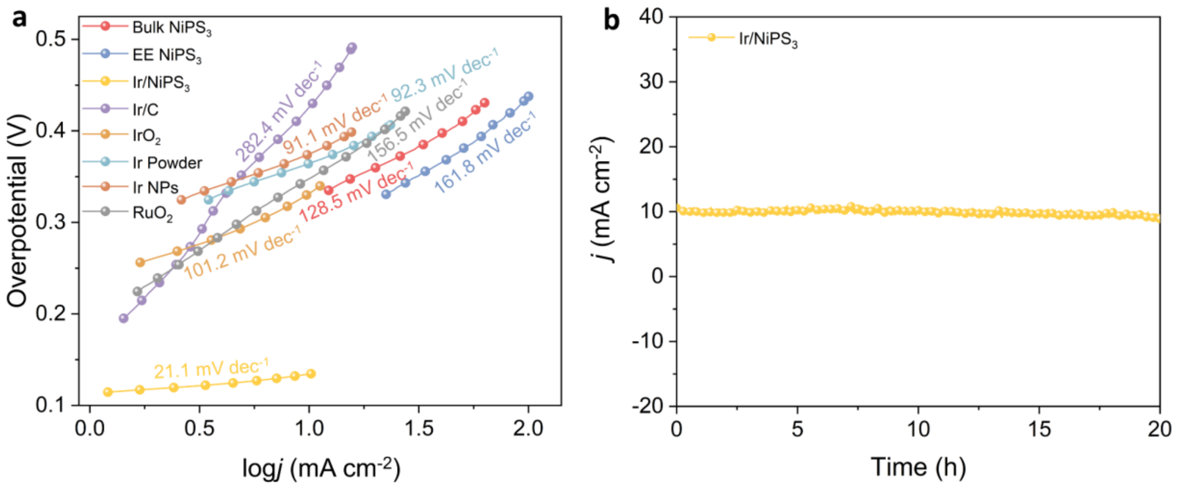

Supplementary Fig. 27. Electrocatalytic performance of samples in 1 M KOH + 0.5 M urea electrolyte for UOR: (a) Tafel plots, (b) Chronopotentiometry curve.

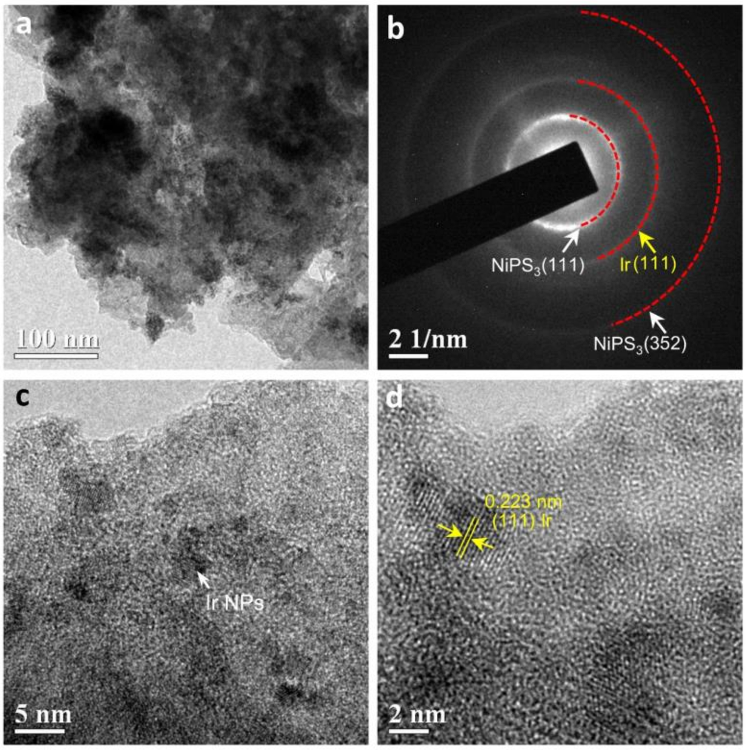

Supplementary Fig. 28. (a) TEM image, (c) SAED pattern, (c,d) HR-TEM image of Ir/$NiPS_3$ after HER cycling test in 1 M KOH + 0.5 M urea.

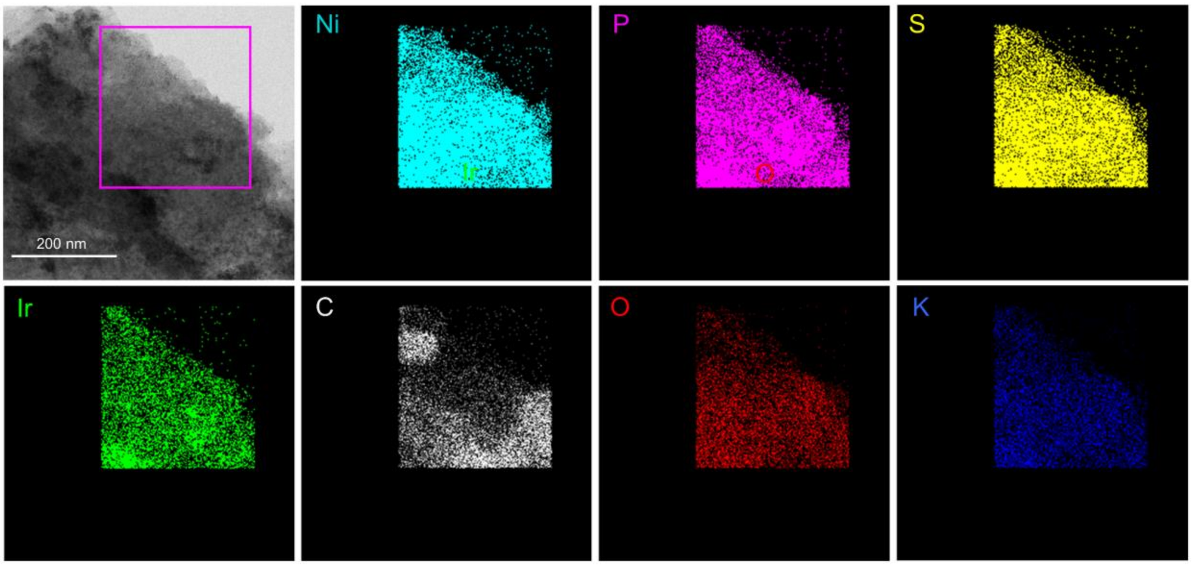

Supplementary Fig. 29. EDS mapping images of Ir/$NiPS_3$ after HER cycling test in 1 M KOH + 0.5 M urea.

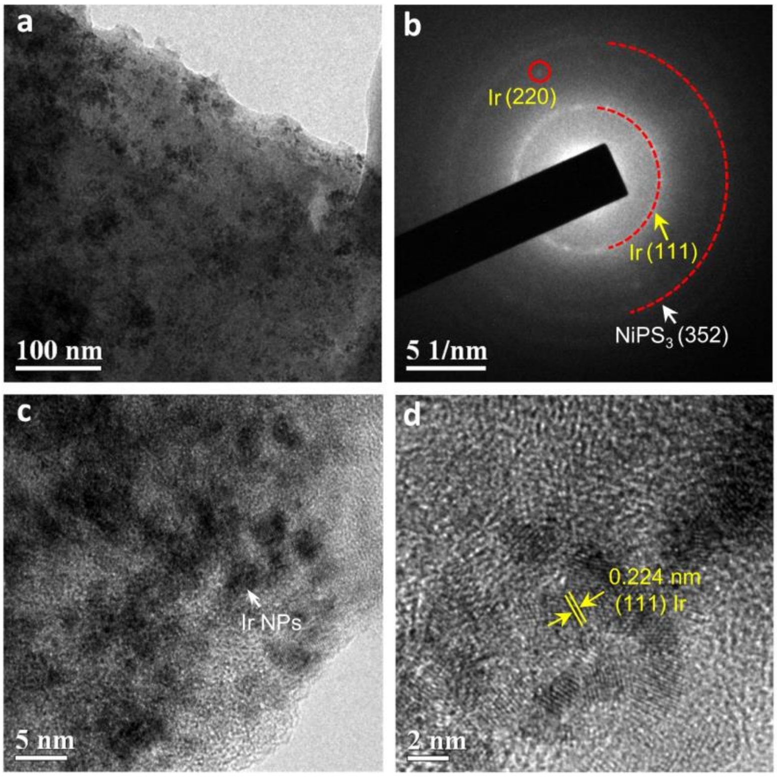

Supplementary Fig. 30. (a) TEM image, (b) SAED pattern, (c,d) HR-TEM image of Ir/$NiPS_3$ after UOR cycling test in 1 M KOH + 0.5 M urea.

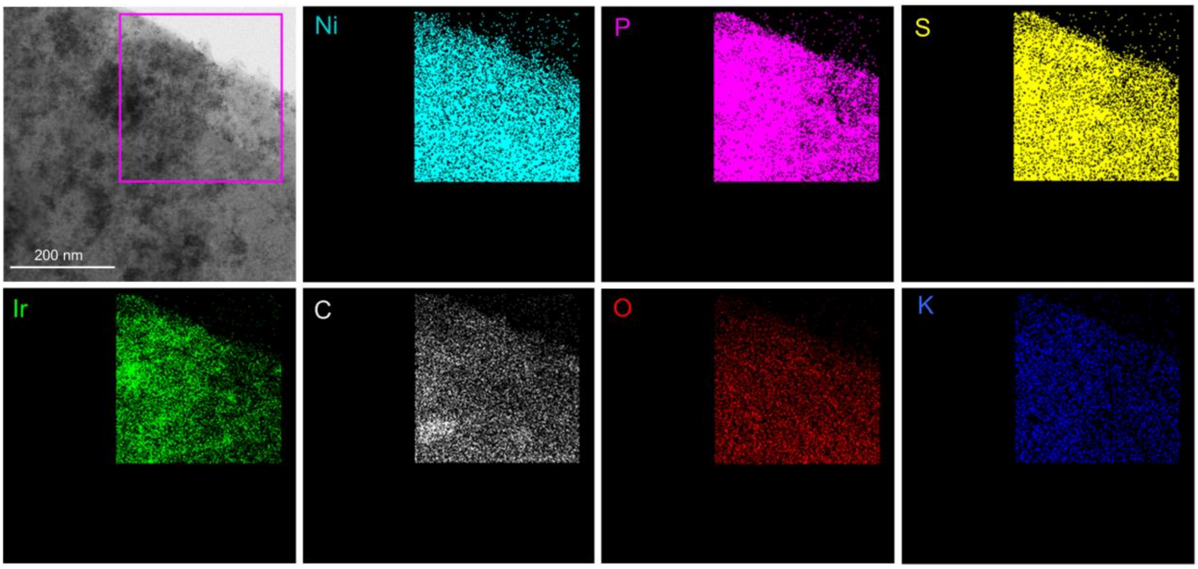

Supplementary Fig. 31. EDS mapping images of Ir/$NiPS_3$ after UOR cycling test in 1 M KOH + 0.5 M urea.

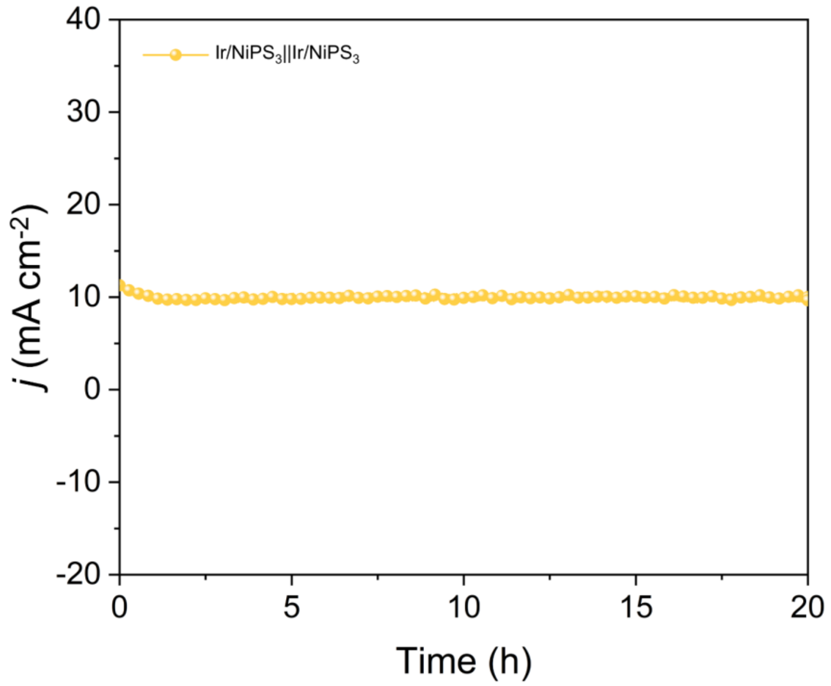

Supplementary Fig. 32. Chronopotentiometry curve for urea-assisted overall water splitting based on Ir/$NiPS_3$||Ir/$NiPS_3$ couple.

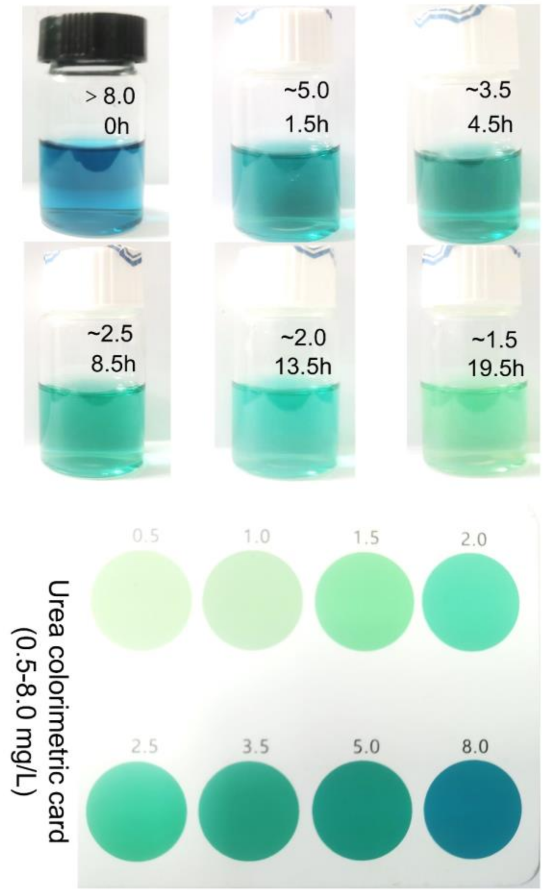

Supplementary Fig. 33. Urea degradation experiment. The 1 × 1 cm^2 nickel foams loaded with Ir/$NiPS_3$ powder were used as the electrode pair for urea degradation. Under constant potential, the urea concentration changed significantly within ~20 hours, and finally reached the water standard. These are the side views of the reagent bottles corresponding to Fig. 4k.

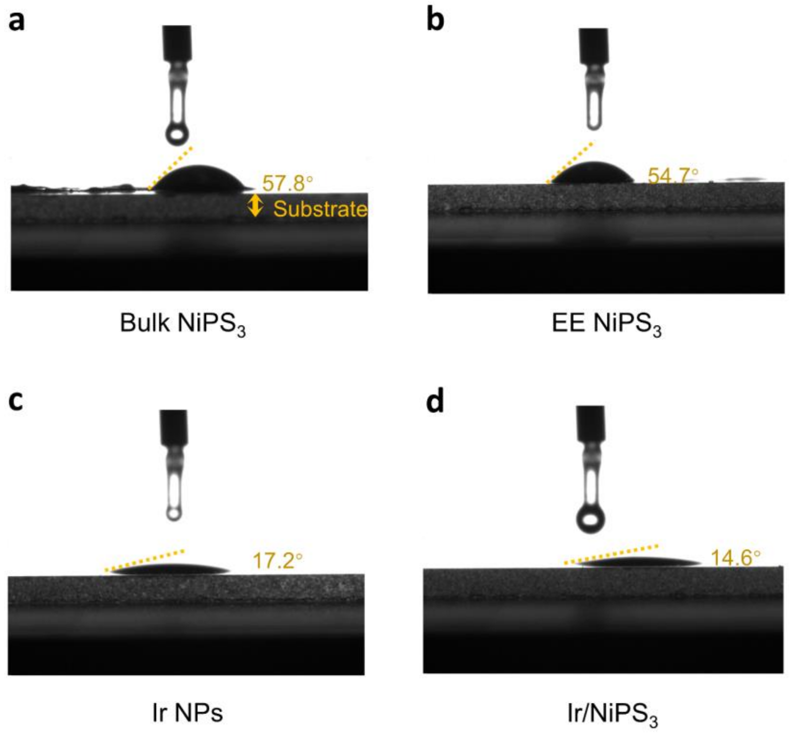

Supplementary Fig. 34. Optical images for contact angles of (a) Bulk $NiPS_3$, (b) EE-$NiPS_3$, (c) Ir NPs, (d) Ir/$NiPS_3$.

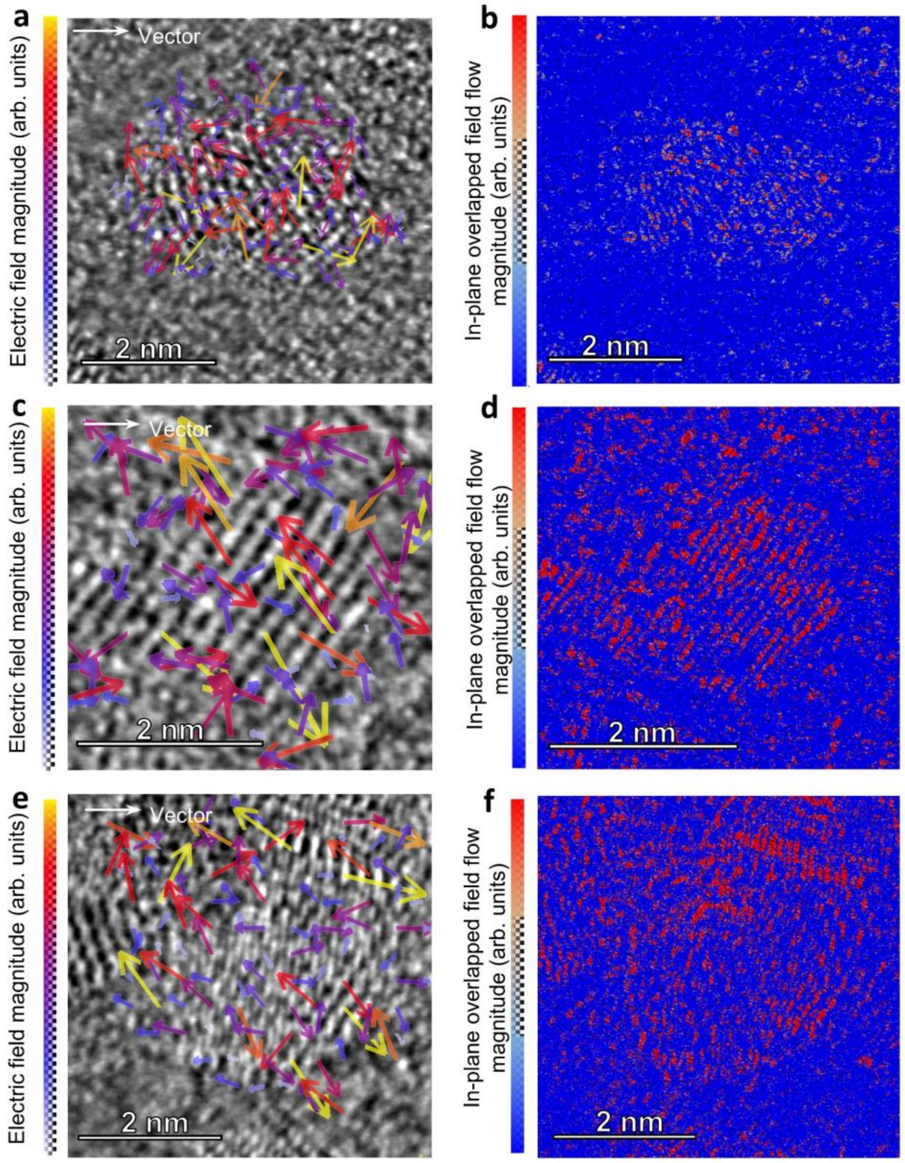

Supplementary Fig. 35. (a,c,e) Differential phase contrast images of the Ir/$NiPS_3$ catalyst. The intensity and direction of the electric field on the surface of the catalyst are represented by an arrow plot. The arrow points in the direction of the electric field, and the color difference represents the intensity of the electric field. (b,d,f) The corresponding plot of the in-plane overlapped field flow magnitude for (a,c,e).

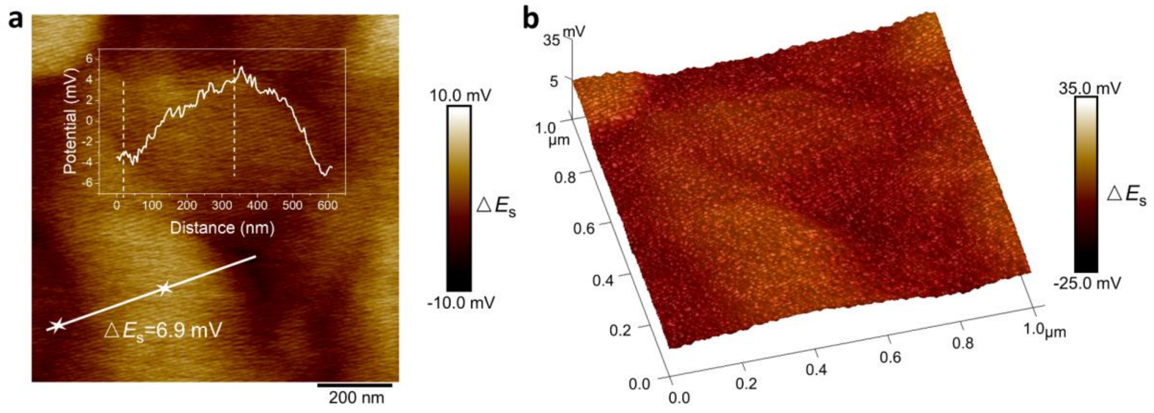

Supplementary Fig. 36. (a) Surface potentials of EE-$NiPS_3$ measured by KPFM and (b) its corresponding 3D diagram.

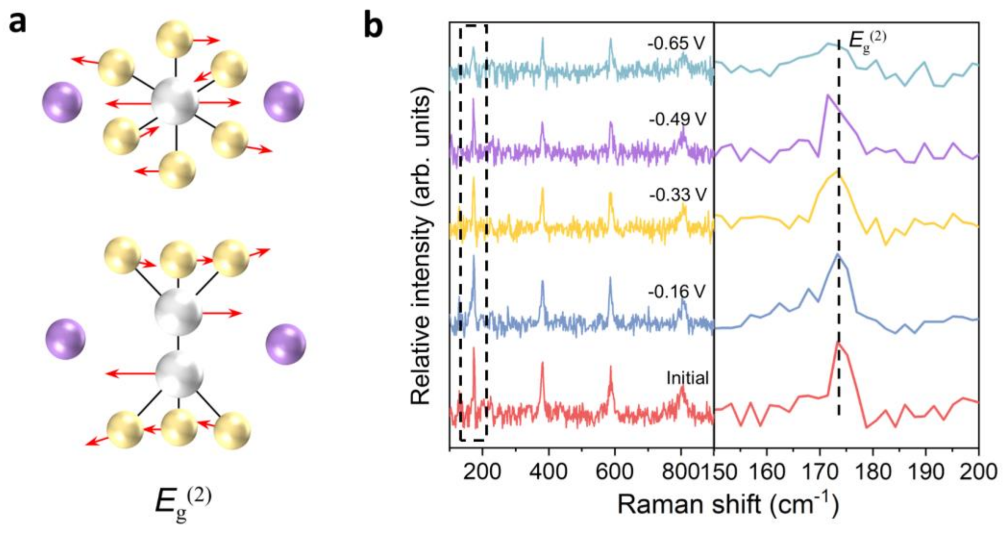

Supplementary Fig. 37. (a) Schematic representation (top view, side view) of vibrational amplitudes of $NiPS_3$ unit cell atoms, for the in-plane $E_g^{(2)}$ phonon modes. (b) The in-situ Raman spectra of $NiPS_3$ against different applied potentials for HER at a range of 100 cm^{-1}-900 cm^{-1}.

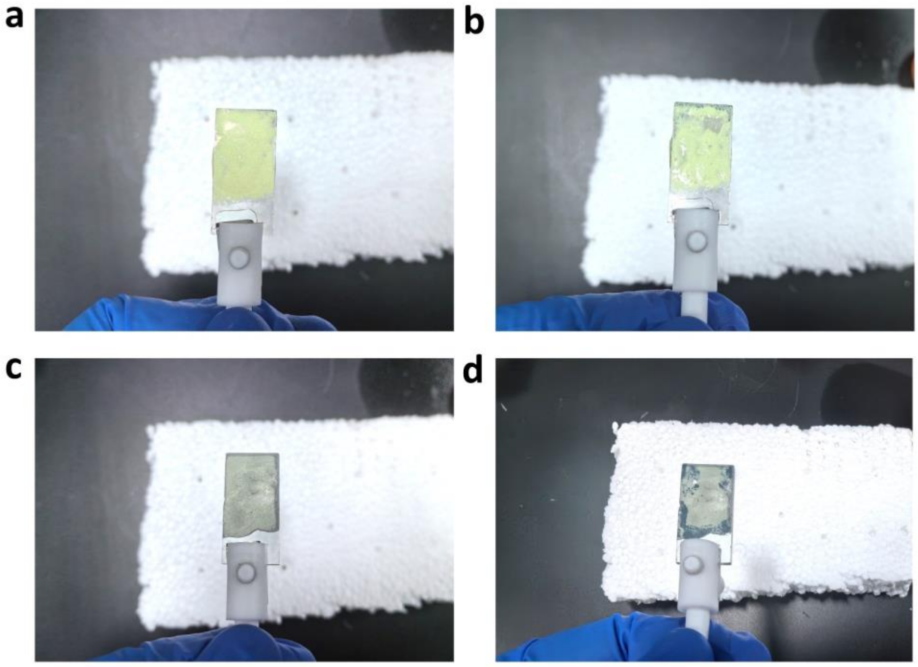

Supplementary Fig. 38. Color change photographs of (a) WO_3, (b) WO_3 after HER, (c) Ir/$NiPS_3$-WO_3 mixture, and (d) Ir/$NiPS_3$-WO_3 mixture after HER. The visual confirmation of the existence of hydrogen spillover was achieved through the color change observed when mixing the Ir/$NiPS_3$ catalyst with WO_3. In an alkaline environment, the color of WO_3 remained unchanged after HER testing (–1.3 V vs. RHE, 5s). However, the mixture of Ir/$NiPS_3$ and WO_3 gradually exhibited a deep blue color after HER testing (–1.3 V vs. RHE, 5s), indicating the occurrence of hydrogen spillover, as the spillover hydrogen migrated and readily reacted with WO_3 to form the deep blue H_xWO_3.

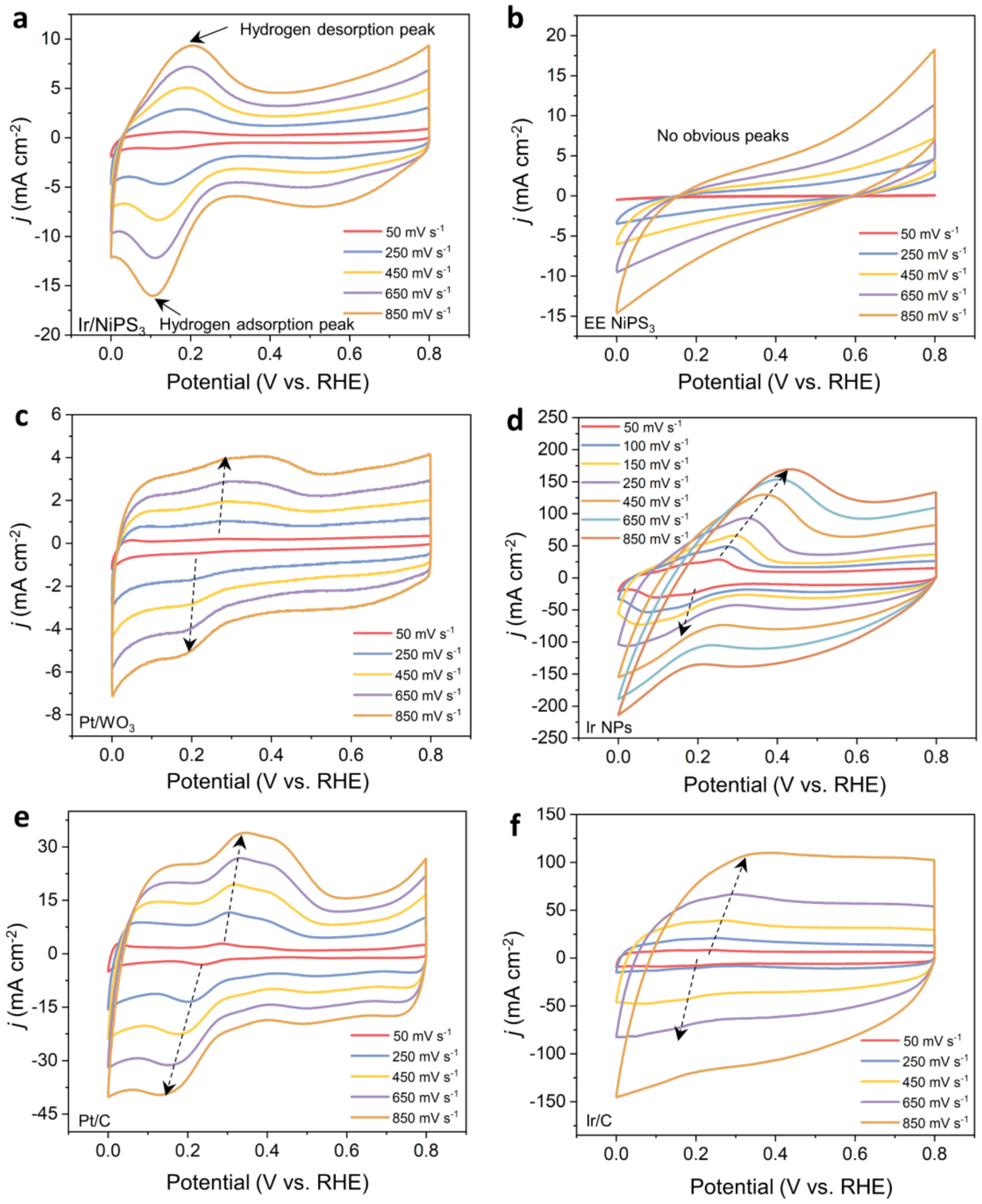

Supplementary Fig. 39. CV profiles of (a) Ir/NiPS$_3$, (b) EE NiPS$_3$, (c) Pt/WO$_3$, (d) Ir NPs, (e) Pt/C and (f) Ir/C catalysts with the scan rate from 50 to 850 mV s^{-1} in 1 M KOH. The catalytic systems' hydrogen adsorption and desorption kinetics were analyzed by in-situ cyclic voltammetry (in-situ CV) to support the occurrence of hydrogen spillover. Hydrogen adsorption peaks and hydrogen desorption peaks were monitored for Ir/NiPS$_3$, NiPS$_3$, as well as the typical hydrogen spillover material Pt/WO$_3$, and the non-hydrogen spillover system Ir NPs. The peaks shifted with variations in the scan rate (Supplementary Fig. 39). NiPS$_3$ showed no clear adsorption and desorption peaks, indicating its weaker hydrogen adsorption capability.

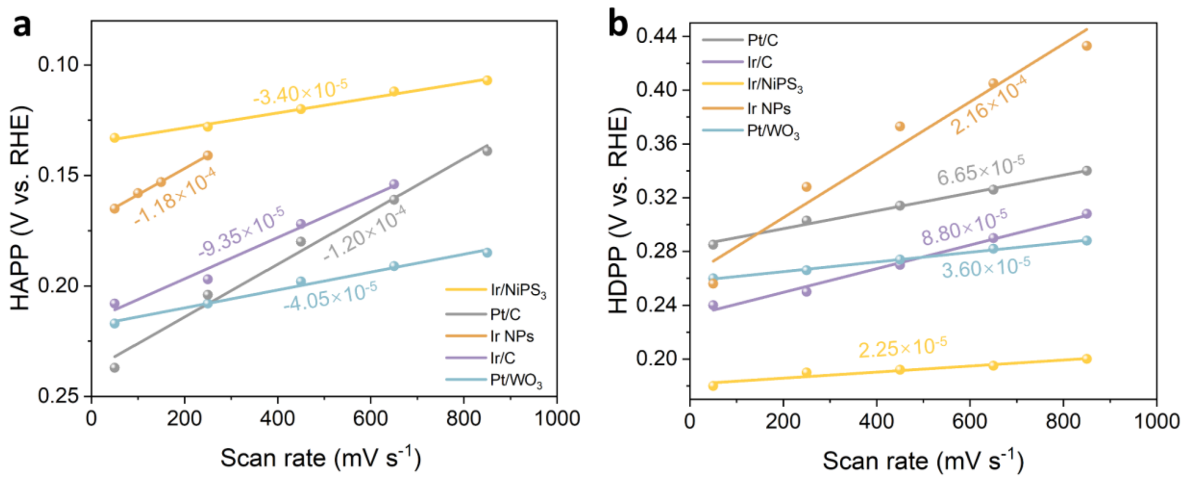

Supplementary Fig. 40. Plots of (a) hydrogen adsorption peak position and (b) hydrogen desorption peak position vs. scan rates. The relationship between the hydrogen adsorption peak position (HAPP) and the hydrogen desorption peak position (HDPP) with the scan rate demonstrated that Ir/$NiPS_3$ exhibited a lower absolute slope value similar to Pt/WO_3 and significantly lower than Ir NPs (Supplementary Fig. 40), indicating accelerated hydrogen adsorption and desorption kinetics. This accelerated hydrogen adsorption/desorption kinetics may stem from the efficient hydrogen spillover effect.

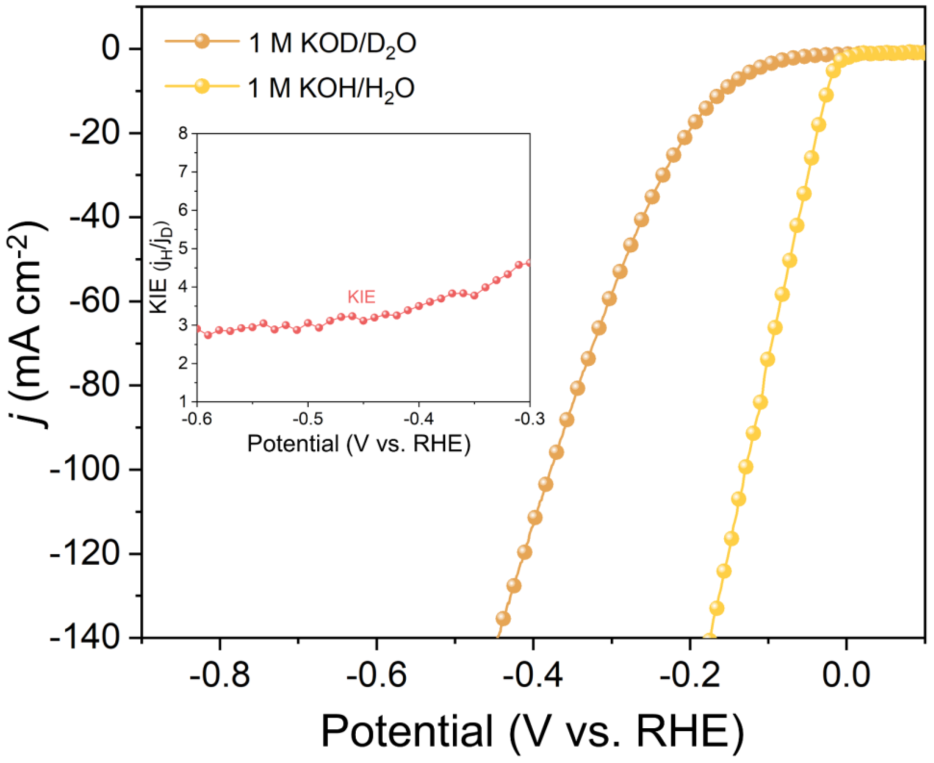

Supplementary Fig. 41. Polarization curves of Ir/$NiPS_3$ catalyst in aqueous 1 M KOH/H_2O solution and 1 M KOD/D_2O solution. The inset is the kinetic isotope effect value vs. potential. In addition to hydrogen adsorption and desorption, in-situ Raman spectroscopy provides evidence of hydrogen migration (Fig. 5f). Besides, the hydrogen/deuterium (H/D) kinetic isotope effects (KIEs) can reflect the kinetic information of hydrogen or proton transfer in chemical reactions. Compared to the polarization curve of Ir/$NiPS_3$ in 1 M KOH/H_2O solution, the current density in 1 M KOD/D_2O is significantly reduced by approximately 2.7-4.6 times across the entire potential range (KIEs = 2.7-4.6) (Supplementary Fig. 41). The presence of KIEs (KIEs > 1.5) is considered as crucial evidence that proton or hydrogen transfer is a key factor affecting reaction rate, confirming the possible existence of hydrogen spillover.

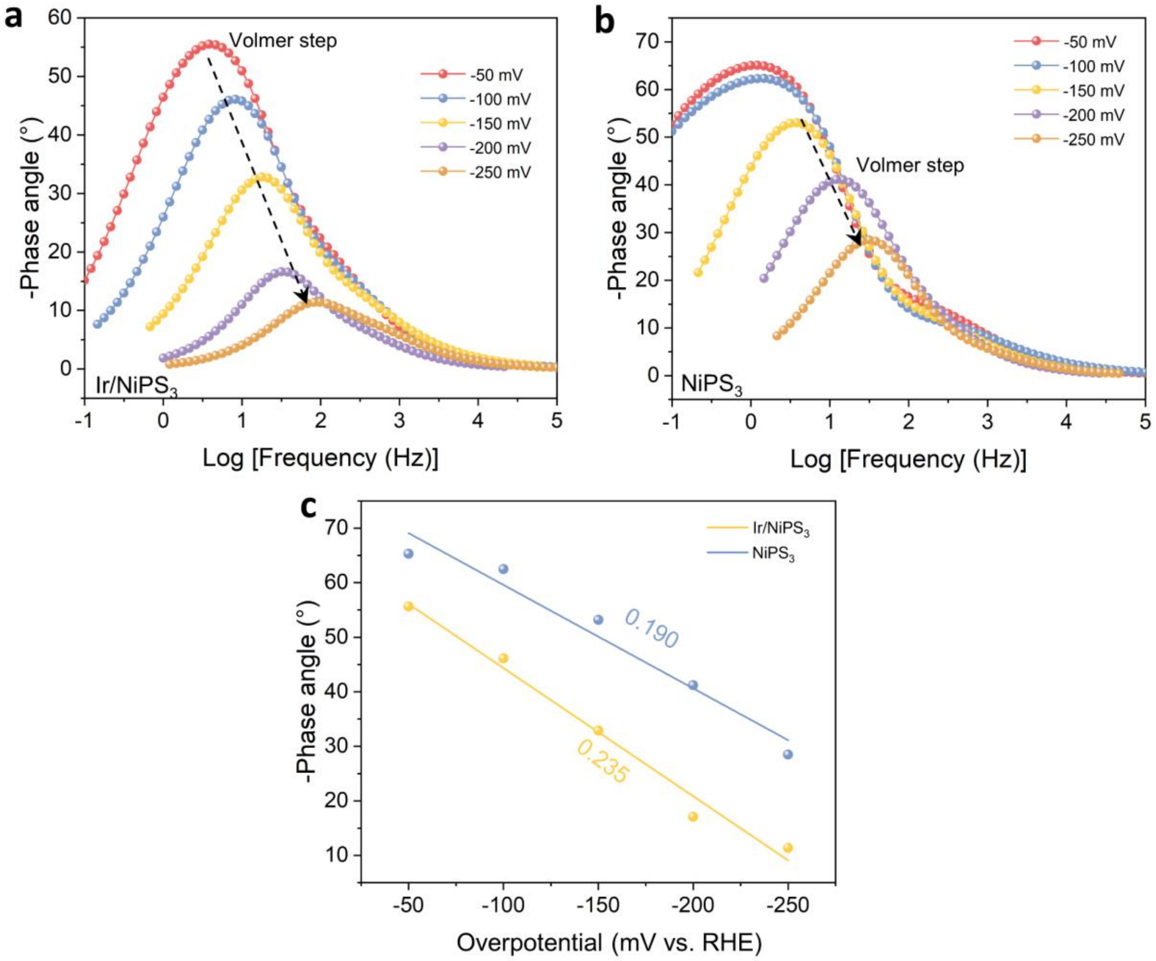

Supplementary Fig. 42. The Bode plots at different HER overpotentials of (a) Ir/$NiPS_3$ and (b) $NiPS_3$ catalysts obtained by operando EIS testing. (c) The plot of phase angle peak values vs. HER overpotentials. The presence of a single-phase angle in the Bode plot indicates that the HER of the sample follows the Volmer-Tafel mechanism. The only phase angle in the Supplementary Fig. 42a,b represent the Volmer process (water dissociation, $*+H_2O+e^- = *H+OH^-$). The slope of Ir/$NiPS_3$ is greater than pure $NiPS_3$ (Supplementary Fig. 42c), and the phase angle peak value rapidly decreases with increasing overpotential for Ir/$NiPS_3$. This demonstrates the enhanced dynamics of the water dissociation step relative to $NiPS_3$.

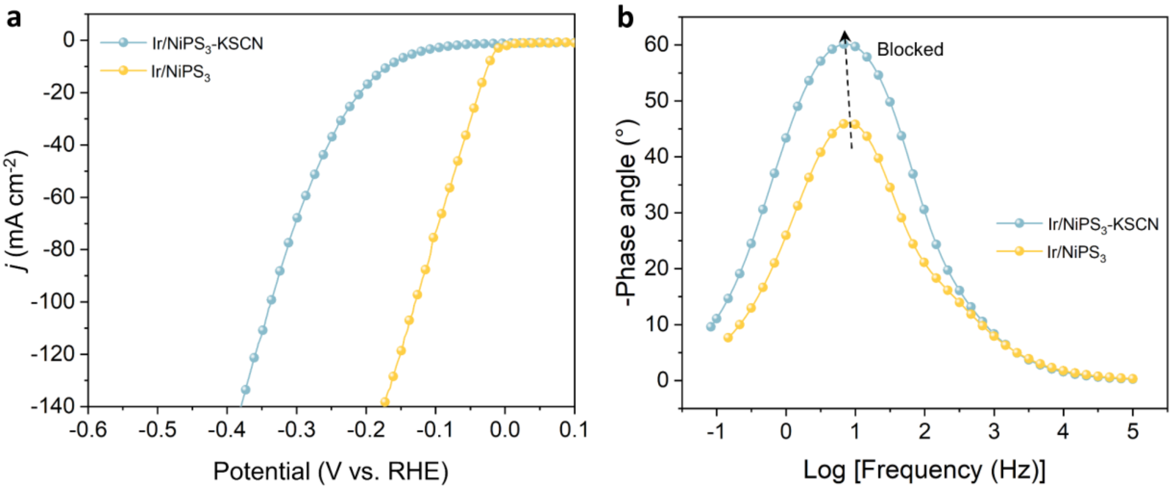

Supplementary Fig. 43. Catalyst poisoning experiment for Ir/$NiPS_3$. (a) LSV plot and (b) Bode plot at an overpotential of –100 mV vs. RHE. Poisoning experiments were conducted using KSCN to inhibit the activity of Ir NPs. In the presence of KSCN, the current density of Ir/$NiPS_3$ was significantly reduced, accompanied by a rapid increase in the phase angle. This indicates that the inhibition of the active sites of Ir NPs reduces the kinetics of water dissociation.

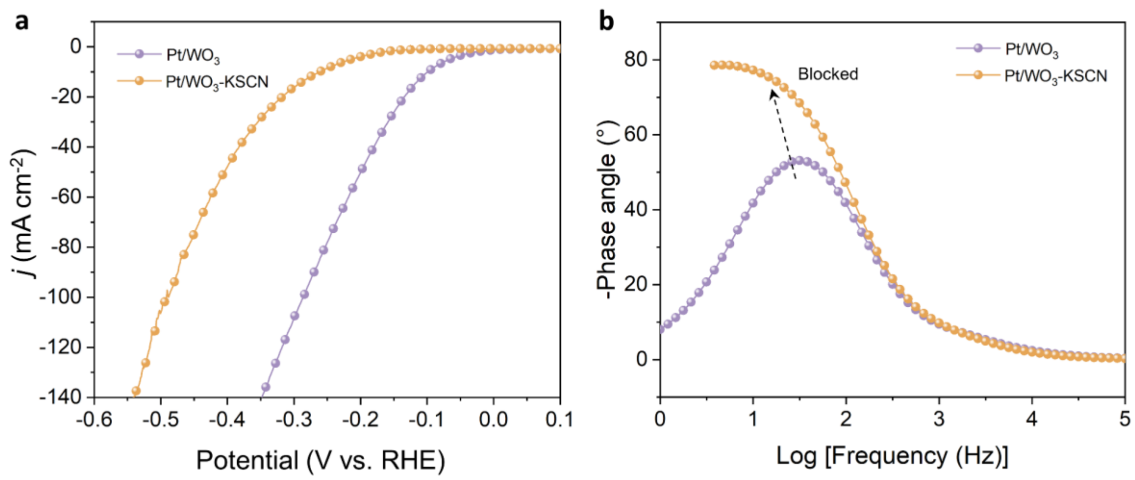

Supplementary Fig. 44. KSCN poisoning experiments for the Pt/WO_3 catalyst. (a) LSV plot and (b) Bode plot at an overpotential of –100 mV vs. RHE.

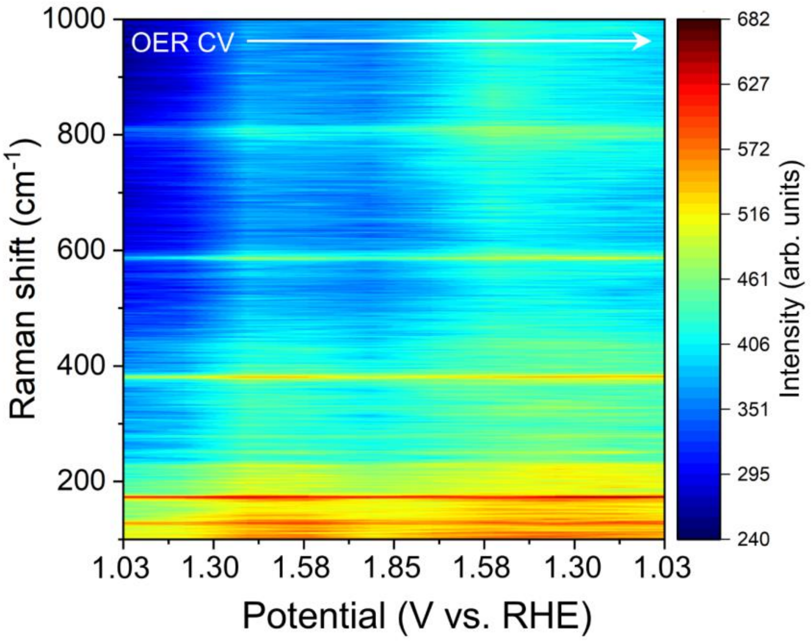

Supplementary Fig. 45. The corresponding 2D top-down projection mapping of Fig. 5g.

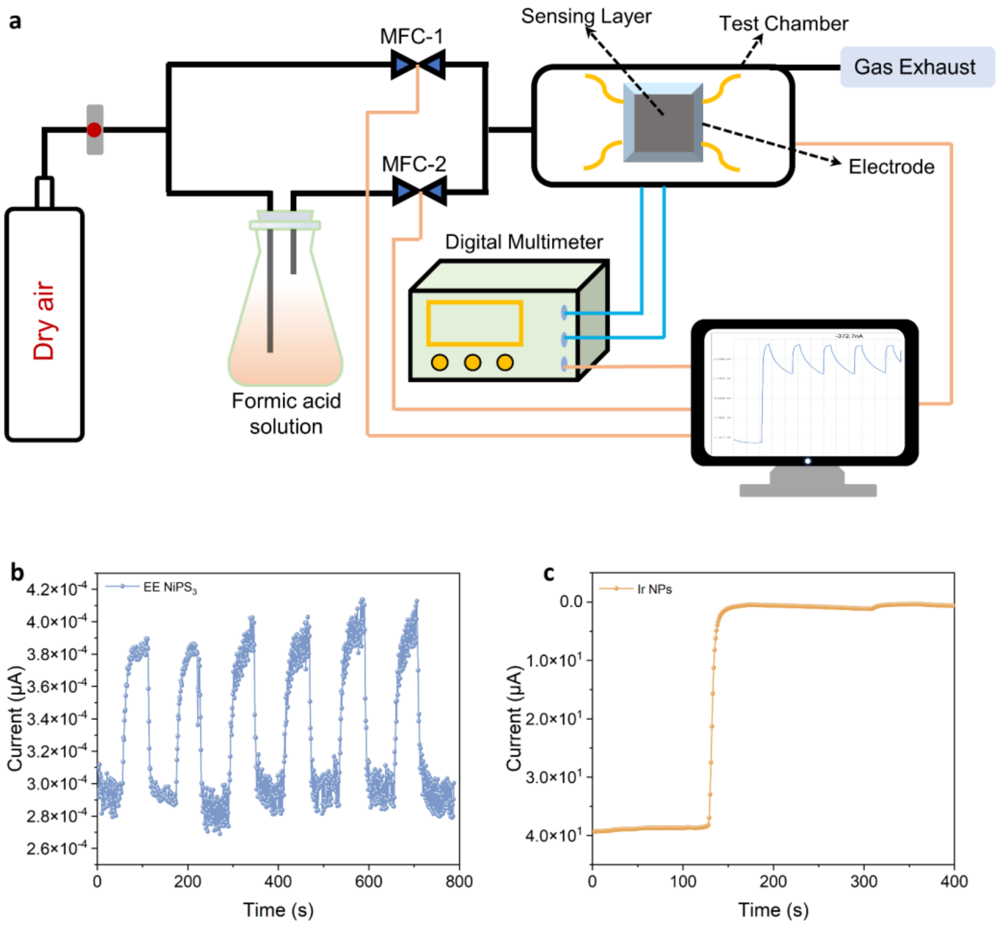

Supplementary Fig. 46. (a) The schematic diagram of the dynamic formic acid sensing test device. Response of (b) EE-$NiPS_3$, (c) Ir NPs when cycled repeatedly between dry air and formic acid vapor.

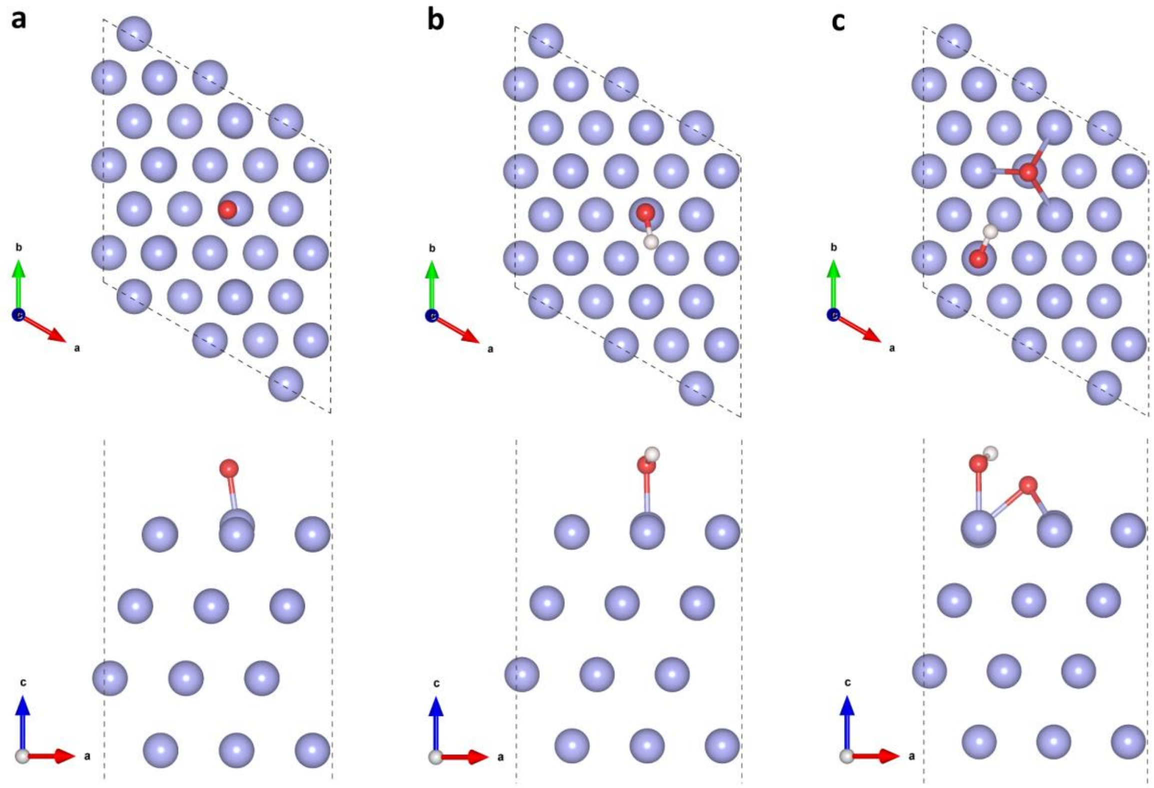

Supplementary Fig. 47. Atomic models of Ir NPs absorbed (a) *O, (b) *OH, and (c) *OOH. *OOH intermediates will break bonds to form *O and *OH in the optimization process, so it is not conducive to the continuation of OER process.

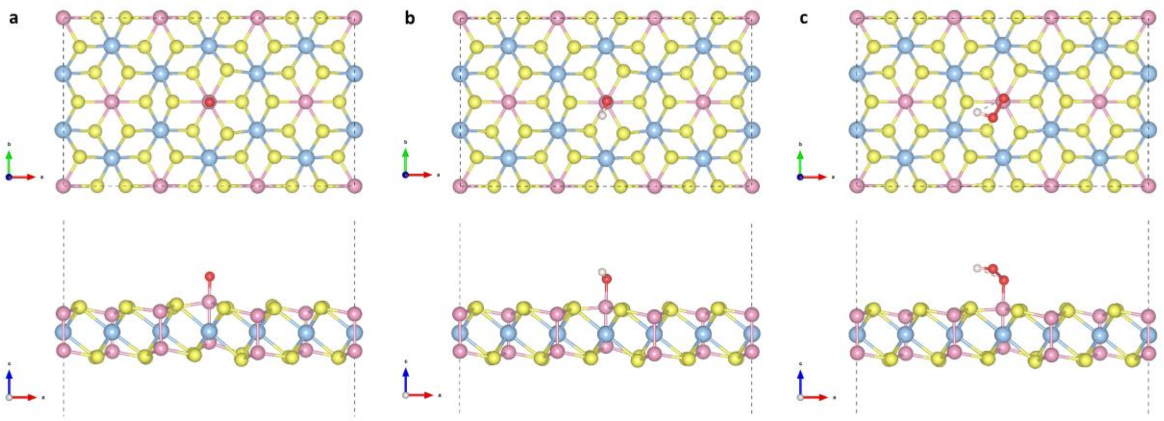

Supplementary Fig. 48. Atomic models of $NiPS_3$ absorbed (a) *O, (b) *OH, and (c) *OOH on P site.

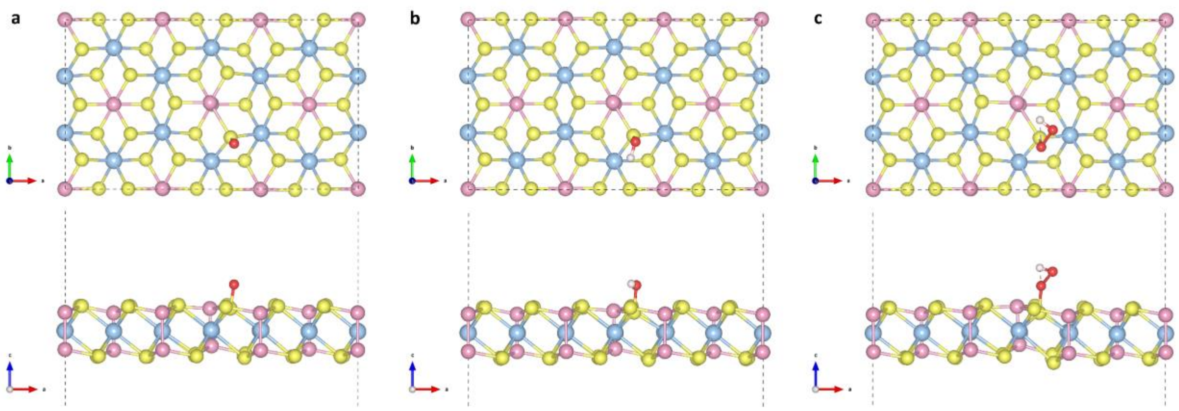

Supplementary Fig. 49. Atomic models of $NiPS_3$ absorbed (a) *O, (b) *OH, and (c) *OOH on S site.

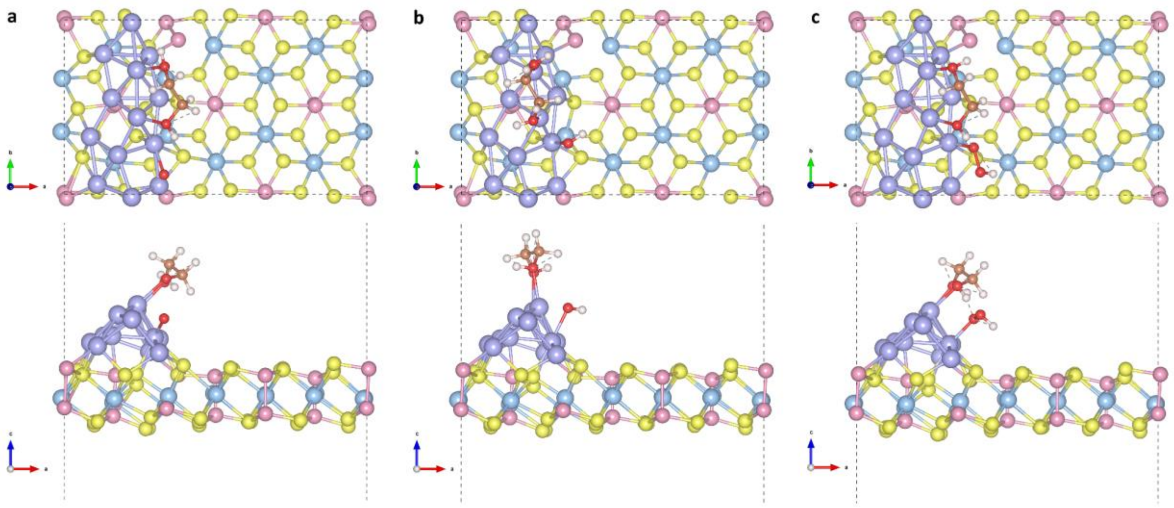

Supplementary Fig. 50. Atomic models of Ir/$NiPS_3$ absorbed (a) *O, (b) *OH, and (c) *OOH on Ir site.

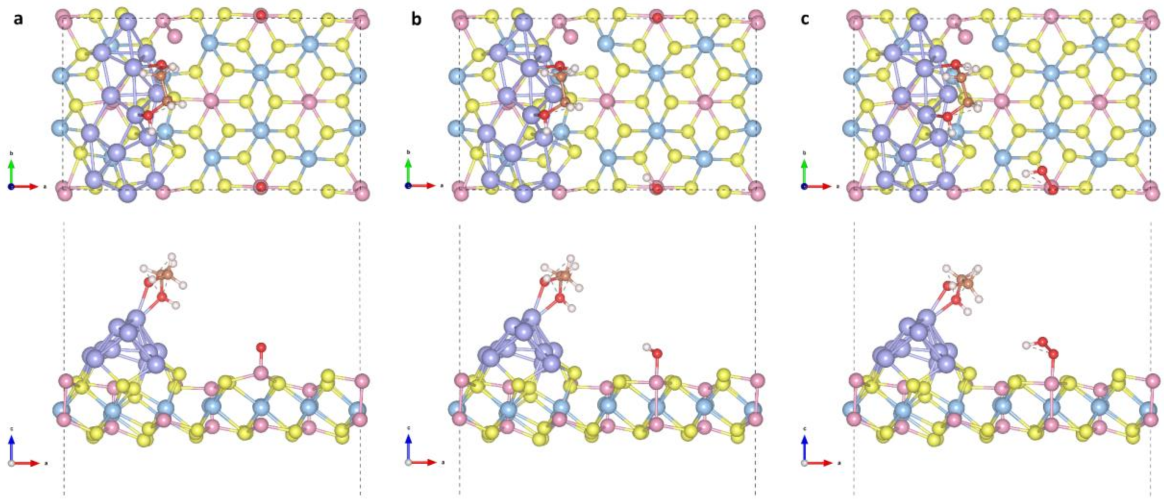

Supplementary Fig. 51. Atomic models of Ir/$NiPS_3$ absorbed (a) *O, (b) *OH, and (c) *OOH on P site.

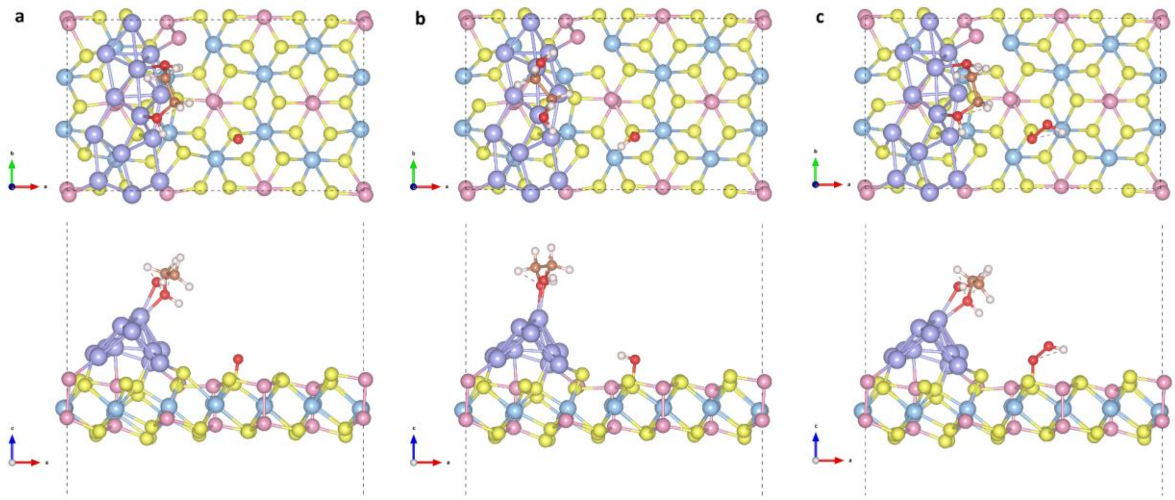

Supplementary Fig. 52. Atomic models of Ir/$NiPS_3$ absorbed (a) *O, (b) *OH, and (c) *OOH on S site.

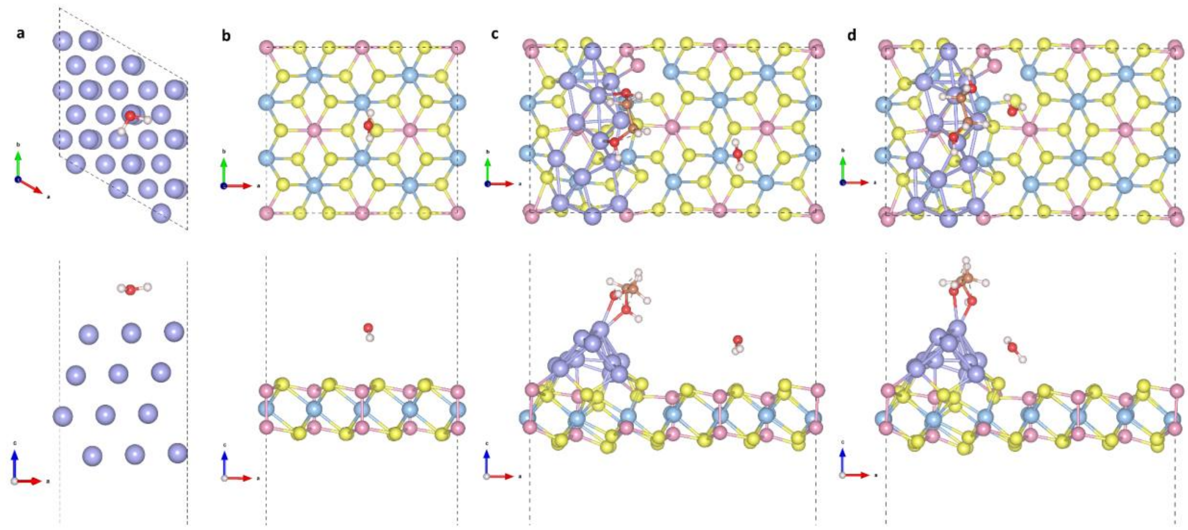

Supplementary Fig. 53. Atomic models of H_2O adsorption on (a) Ir, (b) $NiPS_3$, (c) base plane of Ir/$NiPS_3$, and (d) Ir side of Ir/$NiPS_3$.

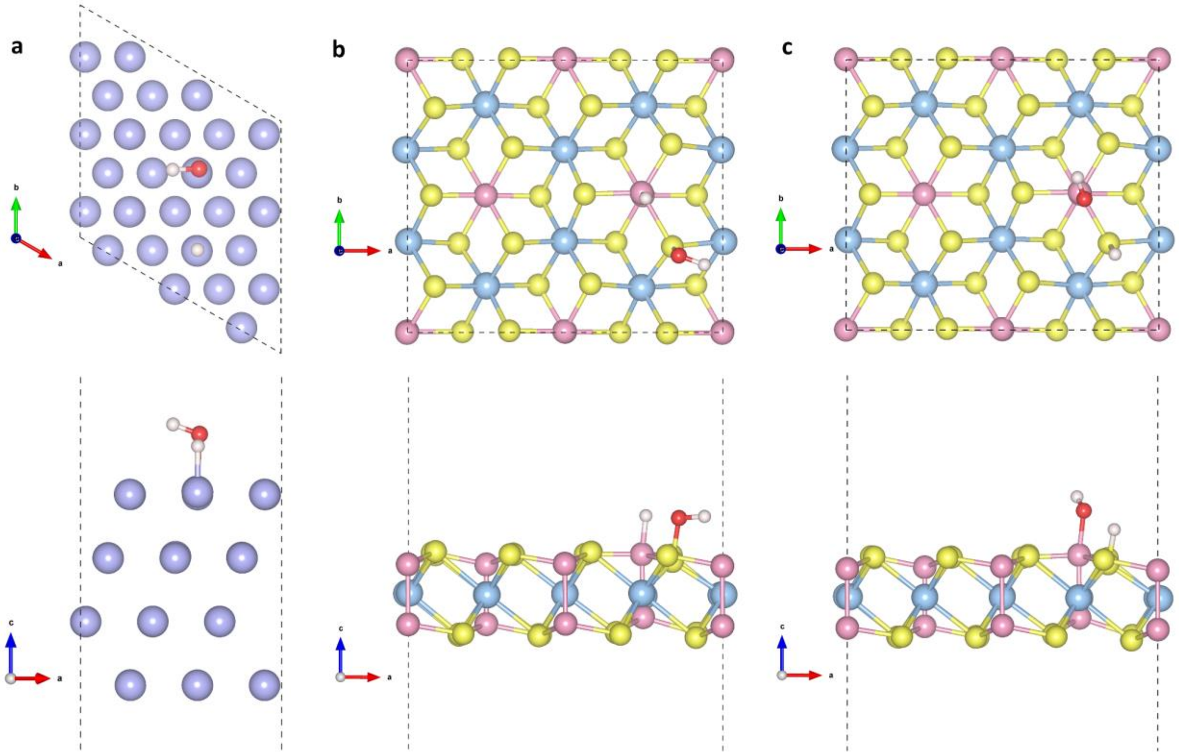

Supplementary Fig. 54. The possible water dissociation and H-OH stable adsorption sites on (a) Ir Ir site, (b) $NiPS_3$ S site, and (c) $NiPS_3$ P site. We stipulate that the adsorption positions of *OH after dissociation are used as the basis for describing the adsorption site.

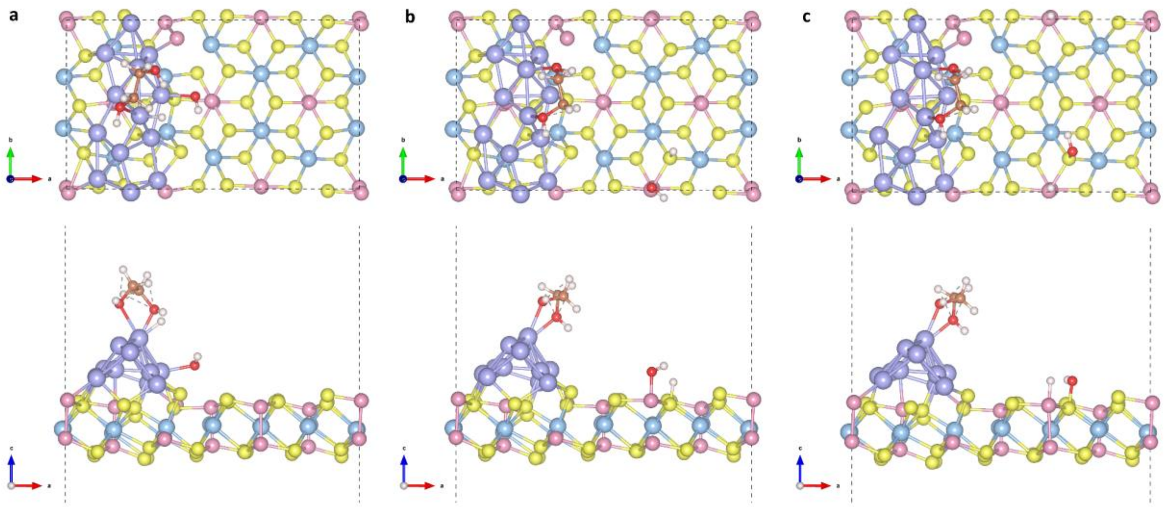

Supplementary Fig. 55. The possible water dissociation and H-OH stable adsorption sites on Ir/$NiPS_3$ (a) Ir site, (b) P site, and (c) S site. We stipulate that the adsorption positions of *OH after dissociation are used as the basis for describing the adsorption site.

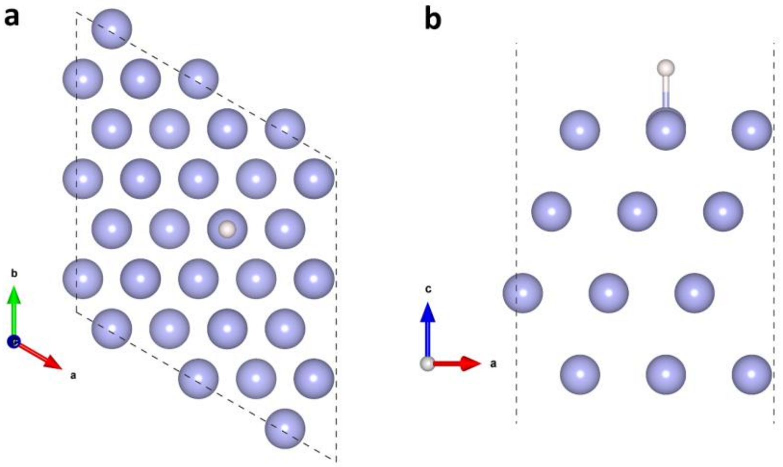

Supplementary Fig. 56. (a) Top and (b) side view for the atomic model of *H adsorption on Ir surface.

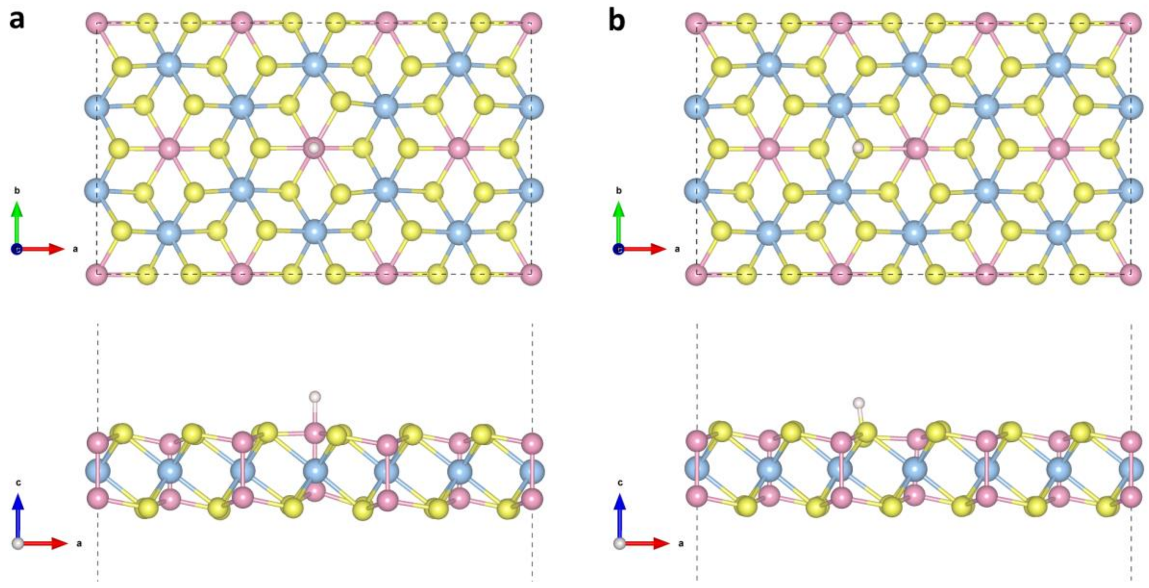

Supplementary Fig. 57. The atomic models of *H adsorption on $NiPS_3$ (a) P and (b) S site.

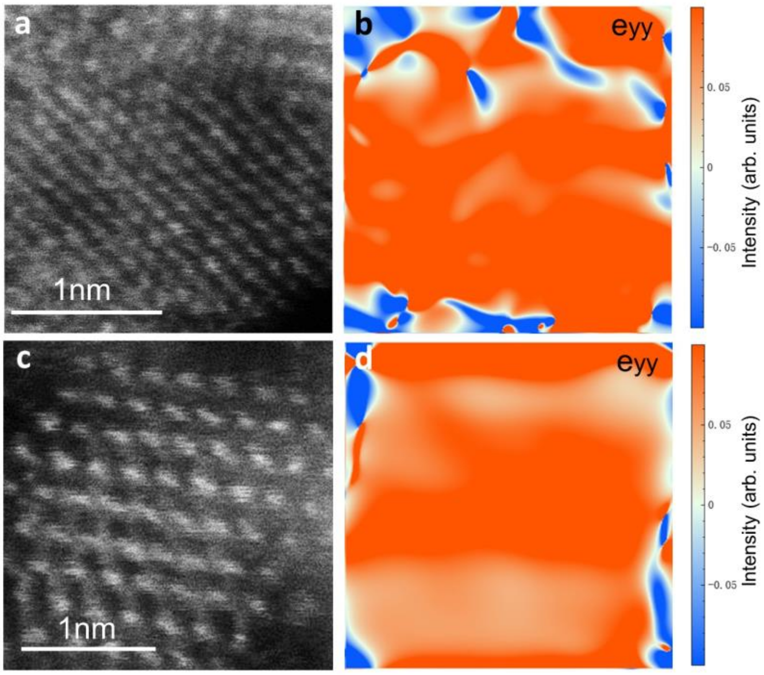

Supplementary Fig. 58. (a,c) The HADDF-STEM image and (b,d) corresponding e_{yy} strain component of the crystalline domain for (a) and (c).

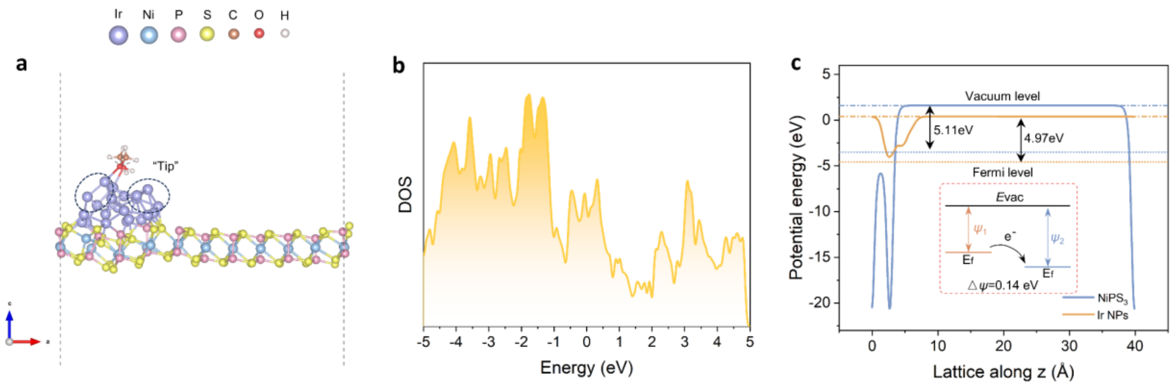

Supplementary Fig. 59. (a) The atomic model and (b) DOS of expanded Ir/$NiPS_3$. (c) Work functions of Ir NPs and $NiPS_3$.

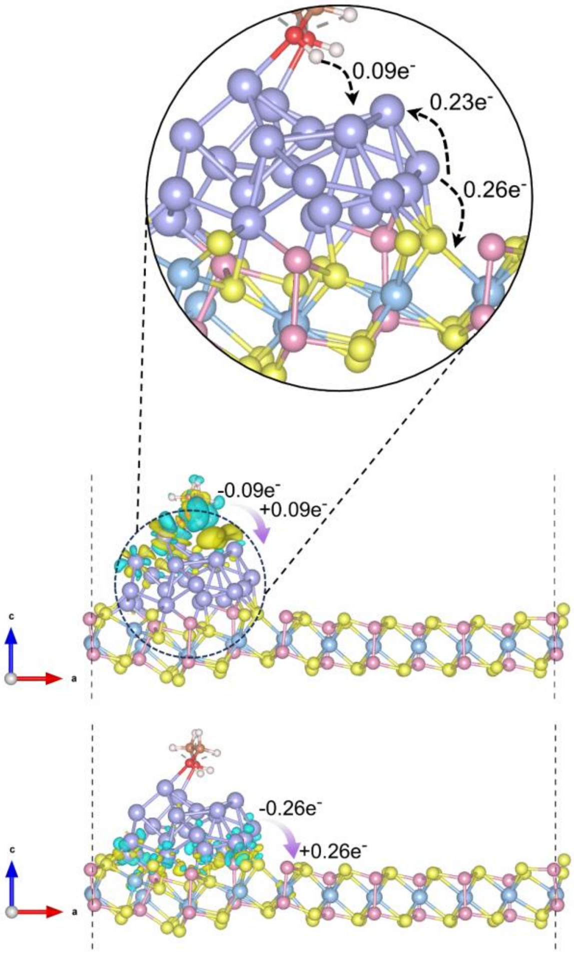

Supplementary Fig. 60. The charge density difference between different parts in expanded Ir/$NiPS_3$ model by Bader charge analysis, showing a clear Janus electronic state.

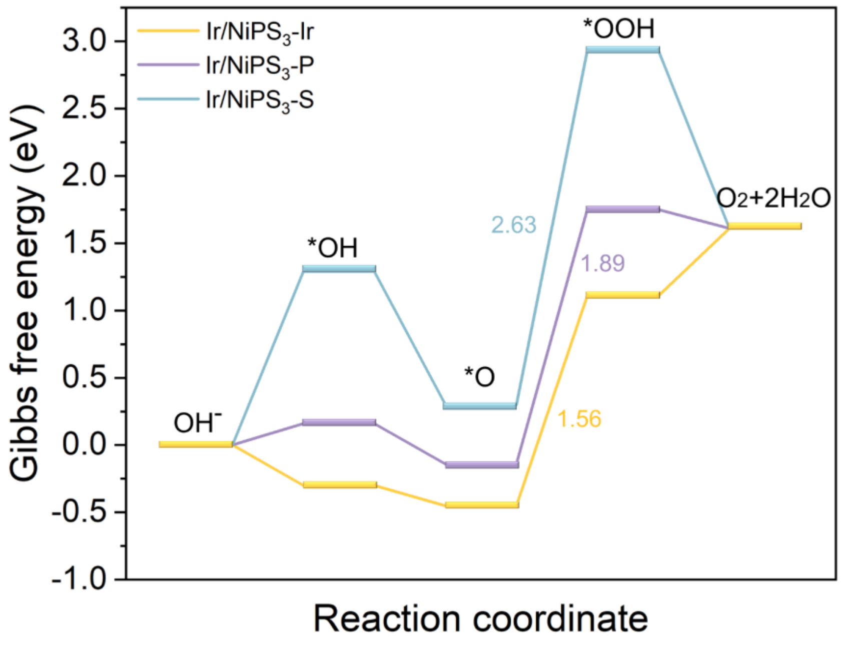

Supplementary Fig. 61. Energy barriers for four-electron-step OER process in the expanded $Ir/NiPS_3$ model. The Ir^{interf} site exhibits the lowest energy barrier, revealing its high activity.

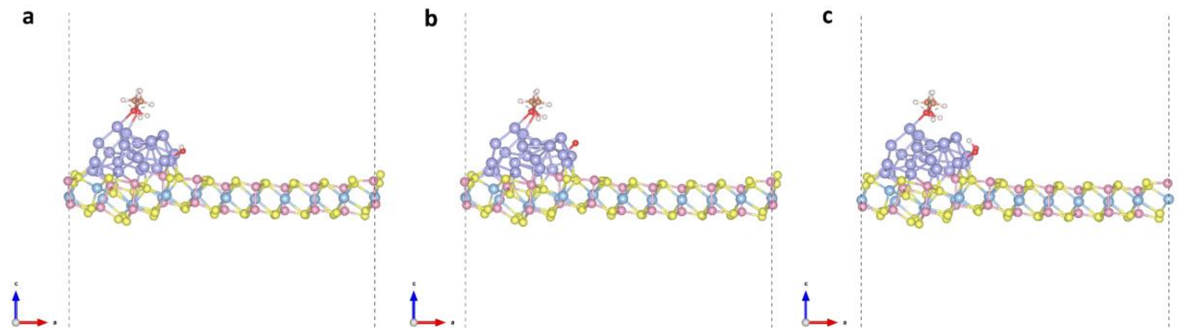

Supplementary Fig. 62. Atomic models of the expanded Ir/$NiPS_3$ model absorbed (a) *OH, (b) *O, and (c) *OOH on Ir site.

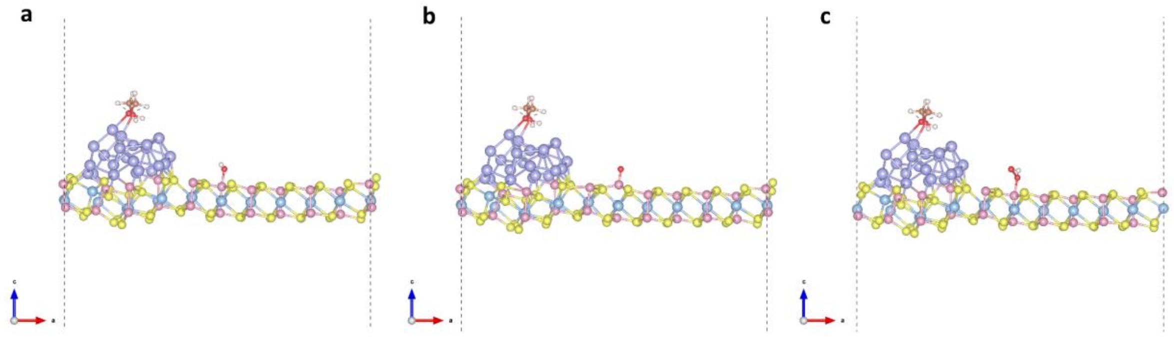

Supplementary Fig. 63. Atomic models of the expanded Ir/$NiPS_3$ model absorbed (a) *OH, (b) *O, and (c) *OOH on P site.

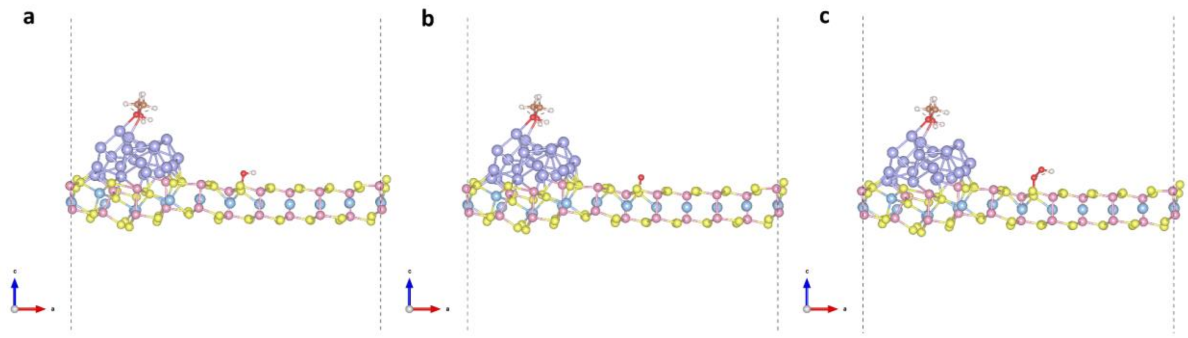

Supplementary Fig. 64. Atomic models of the expanded Ir/$NiPS_3$ model absorbed (a) *OH, (b) *O, and (c) *OOH on S site.

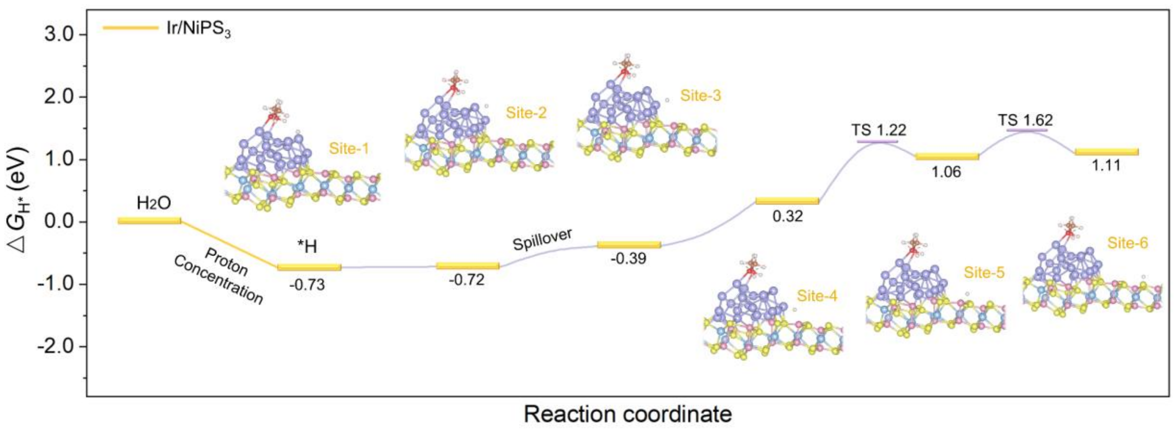

Supplementary Fig. 65. Calculated free energy diagram for hydrogen spillover on the expanded Ir/$NiPS_3$ model.

Supplementary Table 1. Comparison of the HER performance of Ir/$NiPS_3$ with that of representative catalysts of similar elements or structures in 1 M KOH.

Catalysts	Overpotential (η_{10}, mV)	Tafel Slope (mV dec^{-1})	References
Ir1@Co/NC	60	119	8
LN-Ru NPs	33	45	9
D-$IrTe_2$ HNSs	54	32.7	10
PtIr/IrO_x-30 NWs/C	20	38	11
MI-PtZnCo trimetals	29	64.2	12
Pt/TiO_2/$Ni(OH)_2$/NF	26	38.9	13
Pt–IrO_2/CC	22	35	14
$Ir_{0.05}$-Co_2P/$Co_2P_2O_7$ NWs/NF	63	69	15
Ir-g-CN	60.2	49.3	16
FeIr/NF	62	40.94	17
Pt-$Ni(OH)_x$	58	84	18
Ir ONAs	47	111	19
IrCo@NC	45	80	20
NF(Ru-NiFe-P)	44	80	21
IrNi@GO	27	50	22
Ir–Ni thin films	60	40	23
IrP_2@NC	28	50	24
$Ir_{1-x}Rh_xSb$	~200	140	25
Ru@Ni-MOF	22	40	26
$NiRu_{0.13}$-BDC	34	32	27
IrO_2-RuO_2/C	75	48	28
IrO_2/V_2O_5	49	48	29
PdP_2@CB	35.4	42.1	30
Pt/MgO	36	39	31
Pt/MOF-O	66	101.6	32
Pd/$NiFeO_x$/NF	76	78.03	33
Ni-MOF@Pt	102	88	34
AC-Ir NSs	27	26	35
IrNi-N-C	45	50	36
Pt-NC/Ni-MOF	25	42.1	37
Ir/$NiPS_3$ powder	23	32	This work

Supplementary Table 2. Comparison of the OER performance of Ir/$NiPS_3$ with that of representative catalysts of similar elements or structures in 1 M KOH.

Catalysts	Overpotential (η_{10}, mV)	Tafel slope (mV dec^{-1})	References
Ir_1@Co/NC	260	163	8
PtIr/IrO_x-50 NWs/C	266	44	11
PdP_2@CB	270	78.6	30
Li-$IrSe_2$	270	/	38
Ir@Co NSs	273	99	38
Ir-NR/C	296	60.3	39
Ru_1Ni_1-NCNFs	290	/	40
RuCo NPs	296	92.4	41
Ir_1-$Ni(OH)_2$	260	78	42
IrO_2-RuO_2/C	270	79	43
Pt-NC/Ni-MOF	292	/	28
Ir@N-G-750	270	60	37
$Ir_{0.05}$-Co_2P/$Co_2P_2O_7$ NWs/NF	240 (20)	138	44
Ni_5Co_3Mo-OH/NF	304(100)	56.4	15
Ir/$NiPS_3$ powder	236	83.2	This work

Supplementary Table 3. Comparison of the UOR performance of Ir/$NiPS_3$ with that of representative catalysts of similar elements or structures.

Catalysts	UOR potential@10 mA cm^{-2} (V vs. RHE)	Tafel slope (mV dec^{-1})	References
Ni@NCNT-3	1.38	76	45
Ni-Mo nanotube	1.36	22	46
$Ce(OH)_3$@NiFe-LDHs	1.40	30.3	47
Ni-P NG	1.36	13	48
V-Ni_3N/NF	1.361	-	49
NiF_3/Ni_2P@CC	1.36	33	50
NiS@Ni_3S_2	1.37	40	51
NF/NiMoO-Ar	1.37	19	52
Ni@C-250	1.37	43	53
$Ni(OH)_2$-PBA	1.38	80	54
Ni SAs-NC	1.39	42	55
Ni-S-Se/NF	1.38	28	56
Ir/$NiPS_3$ powder	1.36	21.1	This work

Supplementary Table 4. Comparison of the overall water splitting performance of Ir/$NiPS_3$ with that of representative catalysts of similar elements or structures in 1 M KOH.

Catalysts	η_{HER}@10 mA cm^{-2} (mV)	η_{OER}@10 mA cm^{-2} (mV)	Voltage at η_{10} (V)	References
Ir1@Co/NC||Ir1@Co/NC	60	260	1.603	8
$SrIrO_3$||$SrIrO_3$	72	300	1.59	57
Pt-αFe_2O_3/NF||Pt-αFe_2O_3/NF	90	304(50)	1.51	58
Ir-NR/C||Ir-NR/C	42	296	1.57	40
Ir NSs||Ir NSs	50	266	1.57	59
RuCo NSs||RuCo NSs	26	231	1.52	42
$Ni_{0.93}Ir_{0.07}$/rGO||$Ni_{0.93}Ir_{0.07}$/rGO	32.5	271.8	1.55	60
Ru_1Ni_1-NCFs||Ru_1Ni_1-NCFs	35	290	1.56	41
$Ir_{0.05}$-Co_2P/$Co_2P_2O_7$ NWs/NF||$Ir_{0.05}$-Co_2P/$Co_2P_2O_7$ NWs/NF	63	240(20)	1.52	15
IrO_2-RuO_2/C||IrO_2-RuO_2/C	75	270	1.52	28
IrCo@NC||IrCo@NC	82	302	1.62	61
Ir_3-$Ni(OH)_2$/NF||Ir_3-$Ni(OH)_2$/NF	-	-	1.54	62
Ir/Ni_3Fe/rGO||Ir/Ni_3Fe/rGO	36	254	1.574	63
Ir@N-G-750||Ir@N-G-750	43	270	1.51	44
PdIr UNWs/WFG|| PdIr UNWs/WFG	23	290	1.51	64
PtIr/IrO_x-30 NWs/C||PtIr/IrO_x-50 NWs/C	20	266	1.52	11
Ir/$NiPS_3$ powder||Ir/$NiPS_3$ powder	23	236	1.51	This work

Supplementary Table 5. Comparison of the urea-assisted water splitting performance of Ir/$NiPS_3$ with that of representative catalysts of similar elements or structures.

Catalysts	Voltage at η_{10} (V)	References
HC-NiMoS/Ti	1.59	65
Ni_2P/CFC	1.44	66
$Fe_{11.1\%}$-Ni_3S_2	1.46	67
Mo-doped Ni_3S_2	1.45	68
FQD/CoNi-LDH/NF	1.49	69
Ni_3N/$Ni_{0.2}Mo_{0.8}N$/NF	1.487	70
CoMn/$CoMn_2O_4$	1.48	71
Fe-doped NiS-NiS_2	1.55	72
NiF_3/Ni_2P@CC	1.54	50
Mo-doped Ni_3S_2	1.45	73
Ni-Co_9S_8	1.52	74
Ni-S-Se/NF	1.47	56
Ir/$NiPS_3$ powder	1.44	This work

Supplementary Table 6. The calculation values in alkaline OER conditions (calibration at 298.15 K, the unit of the physical quantity is eV, $U = 0$ V).

System	Site	G^*	G_{H2O}	G_{H2}	G_{O2}	$\triangle G_1$	$\triangle G_2$	$\triangle G_3$	$\triangle G_4$	η
Ir	Ir	–322.73	–14.22	–6.8	–9.91	–0.13	0.24	-	-	-
$NiPS_3$	P	–307.82				–0.22	–0.26	1.95	0.14	1.55
	S					1.06	–1.08	2.75	–1.12	2.35
Ir/$NiPS_3$	Ir	–452.82				–0.21	–0.12	1.09	0.85	0.69
	P					–0.06	–0.34	1.94	0.07	1.54
	S					1.03	–0.85	2.50	–1.07	2.10

Supplementary Table 7. The calculation values of H_2O adsorption on the surface of the catalysts.

System	$E_{DFT}^{H_2O^*}$ (eV)	E_{DFT}^{*} (eV)	$E_{DFT}^{H_2O}$ (eV)	ΔE_{H_2O} (eV)
Ir	–337.48	–322.73	–14.22	–0.53
$NiPS_3$	–219.69	–205.33		–0.14
Ir/$NiPS_3$-substrate	–467.31	–452.82		–0.27
Ir/$NiPS_3$-Ir side	–467.66			–0.62

Supplementary Table 8. The calculation values of HER for $\Delta E_{H\text{-}OH}$.

System	OH-Site	$E_{cat.\text{-}(H\text{-}OH)}$ (eV)	$E_{cat.}$ (eV)	E_{H_2O} (eV)	$\Delta E_{H\text{-}OH}$(eV)
Ir	Ir	–336.97	–322.73	–14.22	0.51
$NiPS_3$	P	–218.91	–205.33		0.78
	S	–217.85			1.84
Ir/$NiPS_3$	P	–466.13	–452.82		1.18
	S	–465.20			2.11
	Ir	–467.63			0.03

Supplementary Table 9. The calculation values for HER.

System	Site	$E_{\mathrm{DFT}}^{\mathrm{H}^*}$(eV)	E_{DFT}^{*}(eV)	$E_{\mathrm{DFT}}^{\mathrm{H_2(g)}}$ (eV)	ΔE_{H^*}(eV)	ΔG_{H^*}(eV)
Ir	Ir	–326.57	–322.73	–6.8	–0.44	–0.26
$NiPS_3$	P	–310.85	–307.82		0.37	0.61
	S	–310.53			0.69	0.90
$Ir/NiPS_3$	1	–456.86	–452.82		–0.64	–0.47
	2	–456.49			–0.27	–0.10
	3	–456.29			–0.07	0.09
	4	–456.30			–0.08	0.14
	5	–456.03			0.19	0.41
	6	–455.46			0.76	0.98

Supplementary Table 10. The calculation values for the expanded Ir/$NiPS_3$ model in alkaline OER conditions (calibration at 298.15 K, the unit of the physical quantity is eV, U = 0 V).

System	Site	G^*	G_{H2O}	G_{H2}	G_{O2}	$\triangle G_1$	$\triangle G_2$	$\triangle G_3$	$\triangle G_4$	η
Expanded Ir/$NiPS_3$ model	Ir	–744.71	–14.22	–6.80	–9.91	–0.30	–0.15	1.56	0.50	1.16
	P					0.16	–0.30	1.89	–0.14	1.49
	S					1.29	–0.99	2.63	–1.32	2.23

Supplementary Table 11. The calculation values of HER for the expanded Ir/$NiPS_3$ model.

System	Site	$E_{DFT}^{H^*}$(eV)	E_{DFT}^{*}(eV)	$E_{DFT}^{H_2(g)}$(eV)	ΔE_{H^*}(eV)	ΔG_{H^*}(eV)
Expanded Ir/$NiPS_3$ model	1	–749.00	–744.71	–6.8	–0.89	–0.73
	2	–749.01			–0.90	–0.72
	3	–748.66			–0.55	–0.39
	4	–747.93			0.18	0.32
	5	–747.26			0.85	1.06
	6	–747.20			0.91	1.11

Supplementary References

1. Nørskov, JK. et al. Trends in the exchange current for hydrogen evolution. *J. Electrochem. Soc.* **152**, J23 (2005).
2. Nørskov, JK. et al. Origin of the overpotential for oxygen reduction at a fuel-cell cathode. *J. Phys. Chem. B* **108**, 17886-17892 (2004).
3. Wang, F. et al. Two-dimensional metal phosphorus trisulfide nanosheet with solar hydrogen-evolving activity. *Nano Energy* **40**, 673-680 (2017).
4. Kim, K. et al. Suppression of magnetic ordering in XXZ-type antiferromagnetic monolayer $NiPS_3$. *Nat. Commun.* **10**, 345 (2019).
5. Liu, Y. et al. Synergizing hydrogen spillover and deprotonation by the internal polarization field in a MoS_2/$NiPS_3$ vertical heterostructure for boosted water electrolysis. *Adv. Mater.* **34**, 2203615 (2022).
6. Li, J. et al. Ethylene-glycol ligand environment facilitates highly efficient hydrogen evolution of Pt/CoP through proton concentration and hydrogen spillover. *Energy Environ. Sci.* **12**, 2298-2304 (2019).
7. Braglia, M. et al. Bottom-up electrochemical deposition of poly (styrene sulfonate) on nanoarchitectured electrodes. *ACS Appl. Mater. Inter.* **9**, 22902-22910 (2017).
8. Lai, WH. et al. General π-electron-assisted strategy for Ir, Pt, Ru, Pd, Fe, Ni single-atom electrocatalysts with bifunctional active sites for highly efficient water splitting. *Angew. Chem. Int. Ed.* **58**, 11868-11873 (2019).
9. Jiang, R. et al. Non-equilibrium synthesis of stacking faults-abundant Ru nanoparticles towards electrocatalytic water splitting. *Appl. Catal., B* **316**, 121682 (2022).
10. Pi, Y. et al. Selective surface reconstruction of a defective iridium-based catalyst for high-efficiency water splitting. *Adv. Funct. Mater.* **30**, 2004375 (2020).
11. Huang, H. et al. Equilibrated PtIr/IrO_x atomic heterojunctions on ultrafine 1D nanowires enable superior dual-electrocatalysis for overall water splitting. *Small* **18**, 2201333 (2022).
12. Wang, Y.,Lv, H.,Sun, L.,Jia, F&Liu, B. Ordered mesoporous intermetallic trimetals for efficient and pH-universal hydrogen evolution electrocatalysis. *Adv. Energy Mater.* **12**, 2201478 (2022).
13. Kong, A. et al. Robust Pt/TiO_2/$Ni(OH)_2$ nanosheet arrays enable outstanding performance for high current density alkaline water electrolysis. *Appl. Catal., B* **316**, 121654 (2022).

14. Li, L. et al. Electrochemically modifying the electronic structure of IrO_2 nanoparticles for overall electrochemical water splitting with extensive adaptability. *Adv. Energy Mater.* **10**, 2001600 (2020).
15. Austeria, M.,Dao, HT.,Mai, M&Kim, DH. Dual-phase cobalt phosphide/phosphate hybrid interactions via iridium nanocluster interfacial engineering toward efficient overall seawater splitting. *Appl. Catal., B* **327**, 122467 (2023).
16. Yu, Z. et al. Single-atom Ir and Ru anchored on graphitic carbon nitride for efficient and stable electrocatalytic/photocatalytic hydrogen evolution. *Appl. Catal., B* **310**, 121318 (2022).
17. Shen, F. et al. Bimetallic iron-iridium alloy nanoparticles supported on nickel foam as highly efficient and stable catalyst for overall water splitting at large current density. *Appl. Catal., B* **278**, 119327 (2020).
18. Li, Q. et al. Sowing single atom seeds: A versatile strategy for hyper-low noble metal loading to boost hydrogen evolution reaction. *Adv. Energy Mater.* **13**, 2203955 (2023).
19. Wang, C. et al. Synthesis of Ni–Ir nanocages with improved electrocatalytic performance for the oxygen evolution reaction. *ACS Sustainable Chem. Eng.* **5**, 9787-9792 (2017).
20. Jiang, P. et al. Tuning the activity of carbon for electrocatalytic hydrogen evolution via an iridium-cobalt alloy core encapsulated in nitrogen-doped carbon cages. *Adv. Mater.* **30**, 1705324 (2018).
21. Qu, M. et al. Regulating electron density of NiFe-P nanosheets electrocatalysts by a trifle of Ru for high-efficient overall water splitting. *Appl. Catal., B* **263**, 118324 (2020).
22. Gong, S. et al. O species-decorated graphene shell encapsulating iridium-nickel alloy as an efficient electrocatalyst towards hydrogen evolution reaction. *J. Mater. Chem. A* **7**, 15079-15088 (2019).
23. Wu, W. et al. Galvanostatic electrodeposition of thin-film Ir-Ni electrocatalyst on copper foam for HER performance in alkaline electrolyte. *Catal. Lett.* **150**, 1325-1336 (2020).
24. Pu, Z. et al. A universal synthesis strategy for P-rich noble metal diphosphide-based electrocatalysts for the hydrogen evolution reaction. *Energy Environ. Sci.* **12**, 952-957 (2019).

25. Lin, Z. et al. Realizing negatively charged metal atoms through controllable d-electron transfer in ternary $Ir_{1-x}Rh_xSb$ intermetallic alloy for hydrogen evolution reaction. *Adv. Energy Mater.* **12**, 2200855 (2022).
26. Deng, L. et al. Electronic modulation caused by interfacial Ni-O-M (M= Ru, Ir, Pd) bonding for accelerating hydrogen evolution kinetics. *Angew. Chem. Int. Ed.* **60**, 22276-22282 (2021).
27. Sun, Y. et al. Modulating electronic structure of metal-organic frameworks by introducing atomically dispersed Ru for efficient hydrogen evolution. *Nat. Commun.* **12**, 1369 (2021).
28. Samanta, R.,Panda, P.,Mishra, R&Barman, S. IrO_2-modified RuO_2 nanowires/nitrogen-doped carbon composite for effective overall water splitting in all pH. *Energy Fuels* **36**, 1015-1026 (2022).
29. Zheng, X. et al. Strong oxide-support interaction over IrO_2/V_2O_5 for efficient pH-universal water splitting. *Adv. Sci.* **9**, 2104636 (2022).
30. Luo, F. et al. Palladium phosphide as a stable and efficient electrocatalyst for overall water splitting. *Angew. Chem. Int. Ed.* **57**, 14862-14867 (2018).
31. Tan, H. et al. Engineering a local acid-like environment in alkaline medium for efficient hydrogen evolution reaction. *Nat. Commun.* **13**, 2024 (2022).
32. Wang, M. et al. Site-specified two-dimensional heterojunction of Pt nanoparticles/metal–organic frameworks for enhanced hydrogen evolution. *J. Am. Chem. Soc.* **143**, 16512-16518 (2021).
33. Zhang, W. et al. Porous $Pd/NiFeO_x$ nanosheets enhance the pH-universal overall water splitting. *Adv. Funct. Mater.* **31**, 2107181 (2021).
34. Rui, K. et al. Direct hybridization of noble metal nanostructures on 2D metal-organic framework nanosheets to catalyze hydrogen evolution. *Nano Lett.* **19**, 8447-8453 (2019).
35. Wu, G. et al. In-plane strain engineering in ultrathin noble metal nanosheets boosts the intrinsic electrocatalytic hydrogen evolution activity. *Nat. Commun.* **13**, 4200 (2022).
36. Liu, D. et al. Triggering electronic coupling between neighboring hetero-diatomic metal sites promotes hydrogen evolution reaction kinetics. *Nano Energy* **98**, 107296 (2022).
37. Guo, C. et al. Intermediate modulation on noble metal hybridized to 2D metal-organic framework for accelerated water electrocatalysis. *Chem* **5**, 2429-2441 (2019).

38. Zheng, T. et al. Intercalated iridium diselenide electrocatalysts for efficient pH-universal water splitting. *Angew. Chem.* **131**, 14906-14911 (2019).
39. Babu, DD. et al. Atomic iridium@cobalt nanosheets for dinuclear tandem water oxidation. *J. Mater. Chem. A* **7**, 8376-8383 (2019).
40. Luo, F. et al. Iridium nanorods as a robust and stable bifunctional electrocatalyst for pH-universal water splitting. *Appl. Catal., B* **279**, 119394 (2020).
41. Li, M. et al. RuNi nanoparticles embedded in N-doped carbon nanofibers as a robust bifunctional catalyst for efficient overall water splitting. *Adv. Sci.* **7**, 1901833 (2020).
42. Zhu, T. et al. High-index faceted RuCo nanoscrews for water electrosplitting. *Adv. Energy Mater.* **10**, 2002860 (2020).
43. He, Q. et al. Confining high-valence iridium single sites onto nickel oxyhydroxide for robust oxygen evolution. *Nano Lett.* **22**, 3832-3839 (2022).
44. Wu, X. et al. Metal-support interaction boosted electrocatalysis of ultrasmall iridium nanoparticles supported on nitrogen doped graphene for highly efficient water electrolysis in acidic and alkaline media. *Nano Energy* **62**, 117-126 (2019).
45. Zhang, Q. et al. Nitrogen dopants in nickel nanoparticles embedded carbon nanotubes promote overall urea oxidation. *Appl. Catal., B* **280**, 119436 (2021).
46. Zhang, J-Y. et al. Energy-saving hydrogen production coupling urea oxidation over a bifunctional nickel-molybdenum nanotube array. *Nano Energy* **60**, 894-902 (2019).
47. Fan, J&Du, X. Role of Ce in the enhanced performance of the water oxidation reaction and urea oxidation reaction for NiFe layered double hydroxides. *Dalton Trans.* **51**, 8240-8248 (2022).
48. Pei, C. et al. Nanostructured metallic glass in a highly upgraded energy state contributing to efficient catalytic performance. *Adv. Mater.* **34**, 2200850 (2022).
49. Li, R-Q. et al. 3D self-supported porous vanadium-doped nickel nitride nanosheet arrays as efficient bifunctional electrocatalysts for urea electrolysis. *J. Mater. Chem. A* **9**, 4159-4166 (2021).
50. Wang, K. et al. Engineering NiF_3/Ni_2P heterojunction as efficient electrocatalysts for urea oxidation and splitting. *Chem. Eng. J.* **427**, 130865 (2022).
51. Sha, L. et al. A heterogeneous interface on $NiS@Ni_3S_2/NiMoO_4$ heterostructures for efficient urea electrolysis. *J. Mater. Chem. A* **8**, 18055-18063 (2020).
52. Yu, Z-Y. et al. Ni–Mo–O nanorod-derived composite catalysts for efficient alkaline water-to-hydrogen conversion via urea electrolysis. *Energy Environ. Sci.* **11**, 1890-1897 (2018).

53. Wang, J. et al. Ni/NiO heterostructures encapsulated in oxygen-doped graphene as multifunctional electrocatalysts for the HER, UOR and HMF oxidation reaction. *Catal. Sci. Technol.* **11**, 2480-2490 (2021).
54. Xu, H. et al. Transforming carnation-shaped MOF-Ni to Ni-Fe prussian blue analogue derived efficient bifunctional electrocatalyst for urea electrolysis. *ACS Sustainable Chem. Eng.* **8**, 16037-16045 (2020).
55. Jiang, H. et al. Ni single atoms anchored on N-doped carbon nanosheets as bifunctional electrocatalysts for Urea-assisted rechargeable Zn-air batteries. *Appl. Catal., B* **310**, 121352 (2022).
56. Chen, N.,Du, Y-X.,Zhang, G.,Lu, W-T&Cao, F-F. Amorphous nickel sulfoselenide for efficient electrochemical urea-assisted hydrogen production in alkaline media. *Nano Energy* **81**, 105605 (2021).
57. Yu, J. et al. Monoclinic $SrIrO_3$: an easily synthesized conductive perovskite oxide with outstanding performance for overall water splitting in alkaline solution. *Chem. Mater.* **32**, 4509-4517 (2020).
58. Ye, B. et al. Pt (111) quantum dot decorated flower-like αFe_2O_3 (104) thin film nanosheets as a highly efficient bifunctional electrocatalyst for overall water splitting. *J. Mater. Chem. A* **7**, 11379-11386 (2019).
59. Cheng, Z. et al. Partially hydroxylated ultrathin iridium nanosheets as efficient electrocatalysts for water splitting. *Natl. Sci. Rev.* **7**, 1340-1348 (2020).
60. Zhang, S. et al. Facile fabrication of ultrafine nickel-iridium alloy nanoparticles/graphene hybrid with enhanced mass activity and stability for overall water splitting. *J. Energy Chem.* **49**, 166-173 (2020).
61. Zhou, YQ. et al. Atomic cobalt vacancy-cluster enabling optimized electronic structure for efficient water splitting. *Adv. Funct. Mater.* **31**, 2101797 (2021).
62. Tong, Y. et al. Trace iridium engineering on nickel hydroxide nanosheets as high-active catalyst for overall water splitting. *ChemCatChem* **12**, 5720-5726 (2020).
63. Li, Y. et al. Ir nanodots decorated Ni3Fe nanoparticles for boosting electrocatalytic water splitting. *Chem. Eng. J.* **451**, 138548 (2023).
64. Yang, A. et al. High-density growth of ultrafine PdIr nanowires on graphene: reducing the graphene wrinkles and serving as efficient bifunctional electrocatalysts for water splitting. *Nanoscale* **11**, 14561-14568 (2019).

65. Wang, X. et al. Hierarchical coral-like NiMoS nanohybrids as highly efficient bifunctional electrocatalysts for overall urea electrolysis. *Nano Res.* **11**, 988-996 (2018).
66. Zhang, X. et al. Vapour-phase hydrothermal synthesis of Ni_2P nanocrystallines on carbon fiber cloth for high-efficiency H_2 production and simultaneous urea decomposition. *Electrochim. Acta* **254**, 44-49 (2017).
67. Zhu, W. et al. Wet-chemistry topotactic synthesis of bimetallic iron-nickel sulfide nanoarrays: an advanced and versatile catalyst for energy efficient overall water and urea electrolysis. *J. Mater. Chem. A* **6**, 4346-4353 (2018).
68. Xu, H. et al. A branch-like Mo-doped Ni_3S_2 nanoforest as a high-efficiency and durable catalyst for overall urea electrolysis. *J. Mater. Chem. A* **9**, 3418-3426 (2021).
69. Xu, X. et al. Ultrahigh capacitive deionization performance by 3D interconnected MOF-derived nitrogen-doped carbon tubes. *Chem. Eng. J.* **390**, 124493 (2020).
70. Li, R-Q. et al. Hierarchical $Ni_3N/Ni_{0.2}Mo_{0.8}N$ heterostructure nanorods arrays as efficient electrocatalysts for overall water and urea electrolysis. *Chem. Eng. J.* **409**, 128240 (2021).
71. Wang, C. et al. Bimetal Schottky heterojunction boosting energy-saving hydrogen production from alkaline water via urea electrocatalysis. *Adv. Funct. Mater.* **30**, 2000556 (2020).
72. Huang, S. et al. Construction of Fe-doped NiS-NiS_2 heterostructured microspheres via etching prussian blue analogues for efficient water-urea splitting. *Small* **18**, 2106841 (2022).
73. Duman, FD&Forgan, RS. Applications of nanoscale metal-organic frameworks as imaging agents in biology and medicine. *J. Mater. Chem. B* **9**, 3423-3449 (2021).
74. Hu, W. et al. Hierarchical NiCo-layered double hydroxide nanoscroll@PANI nanocomposite for high performance battery-type supercapacitor. *Electrochim. Acta* **338**, 135869 (2020).
